# Characterization of Nanoprecipitated PET Nanoplastics by ^1^H NMR and Impact of Residual Ionic Surfactant on Viability of Human Primary Mononuclear Cells and Hemolysis of Erythrocytes

**DOI:** 10.3390/polym15244703

**Published:** 2023-12-13

**Authors:** Milica Djapovic, Danijela Apostolovic, Vojislava Postic, Tamara Lujic, Vesna Jovanovic, Dragana Stanic-Vucinic, Marianne van Hage, Veselin Maslak, Tanja Cirkovic Velickovic

**Affiliations:** 1 University of Belgrade, Faculty of Chemistry, Studentski trg 12-16, 11158 Belgrade, Serbia; 2 Immunology and Allergy Division, Center for Molecular Medicine, Department of Medicine Solna, Karolinska Institutet, 171 77 Stockholm, Sweden; danijela.apostolovic@ki.se (D.A.); marianne.van.hage@ki.se (M.v.H.); 3 Serbian Academy of Sciences and Arts, Knez Mihajlova 35, 11102 Belgrade, Serbia

**Keywords:** nanoplastic, nanoprecipitation, PET, surfactant, SDS, cytotoxicity, hemolysis

## Abstract

Manufactured nanoplastic particles (NPs) are indispensable for in vitro and in vivo testing and a health risk assessment of this emerging environmental contaminant is needed. The high surface area and inherent hydrophobicity of plastic materials makes the production of NPs devoid of any contaminants very challenging. In this study, we produced nanoprecipitated polyethylene terephthalate (PET) NPs (300 nm hydrodynamic diameter) with an overall yield of 0.76%. The presence of the ionic surfactant sodium dodecyl sulfate (SDS) was characterized by ^1^H NMR, where the relative ratio of NP/surfactant was monitored on the basis of the chemical shifts characteristic of PET and SDS. For a wide range of surfactant/NP ratios (17:100 to 1.2:100), the measured zeta potential changed from −42.10 to −34.93 mV, but with an NP concentration up to 100 μg/mL, no clear differences were observed in the cellular assays performed in protein-rich media on primary human cells. The remaining impurities contributed to the outcome of the biological assays applied in protein-free buffers, such as human red blood cell hemolysis. The presence of SDS increased the NP-induced hemolysis by 1.5% in protein-rich buffer and by 7.5% in protein-free buffer. As the size, shape, zeta potential, and contaminants of NPs may all be relevant parameters for the biological effects of NPs, the relative quantification of impurities exemplified in our work by the application of ^1^H NMR for PET NPs and the ionic surfactant SDS could be a valuable auxiliary method in the quality control of manufactured NPs.

## 1. Introduction

The exponential surge in global plastic production has engendered a multifaceted crisis. Plastic waste is released into the environment and exposed to the action of microorganisms, thermal stress, oxidative processes, photodegradation, and hydrolytic cleavage. This intricate cascade of events culminates in the formation of minuscule fragments, called microplastics (MPs) (<5 mm), and even smaller fragments, called nanoplastics (NPs) (<1000 nm) [1]. Although the empirical footprint of MPs has been documented across various ecosystems, NPs have recently gained a large amount of attention because of their small size; they are more easily taken up by organisms and likely pose higher ecological and health risks than microplastics. The characterization and quantification of NPs faces analytical challenges as there are substantial difficulties in the separation, visualization, and chemical identification of nanoplastics due to their small sizes, and interferences from coexisting substances [1]. The ability of NPs to bioaccumulate and cotransport pollutants in the whole organism is one of the most dangerous aspects of this form of plastic debris, defining a new class of emerging pollutants that is still largely unknown. A recent review comprehensively presented the up-to-date knowledge on the toxicity of NPs to aquatic organisms at various trophic levels including bacteria, plankton (algae), zooplankton, benthos, and nekton (fish), where the adverse effects of sole NPs and the joint effects of NPs with other frequently detected contaminants are summarized systematically [2]. This review underlines that due to the lack of standard reference material for NPs, it is difficult to compare the results obtained from different studies, and even to conclude on the differentiation between marine and fresh water organisms’ response to nanoplastics. In this context, it is essential to have accurate and representative models of NPs to better understand their toxic effects.

Model NPs are a valuable tool needed for hazard and risk assessment estimation, widely used in various in vitro and in vivo biological experiments looking at the different adverse outcome pathways of modeled NPs.

Several methods have been described for model NPs’ manufacturing of different chemical types for the purposes of method development and validation, as well as in vitro toxicity and risk assessment testing [3]. Particularly challenging is achieving the high-quality, low batch-to-batch variation, and uniform size (ideally monodisperse) of the NPs produced. The high surface area and intrinsic hydrophobicity of the NPs make them an ideal vector for hydrophobic compounds, such as pesticides, but also proteins and heavy metals [4]. NPs manufactured for applications in in vitro testing should be devoid of contaminants, particularly those relevant for cell inflammation or survival. 

Many studies investigated the toxicity of NPs mostly with commercially available spherical polystyrene NPs (PS-NPs) of a known size. However, these NPs were not produced for the purpose of reference materials for NPs found in the environment, and contain additives and surfactants, which may account for the toxicity observed in cellular assays, making the interpretation of results challenging [5,6]. The publication by Pikuda et al. [7] was the first study to demonstrate that additives in commercial NPs have toxic effects and may introduce artifacts in toxicity assessments. In addition, manufacturers mainly use surfactants and are often unwilling to provide information on the presence and quantities of remaining surfactant, or about the details of the surface charge density [8]. Heinlaan et al. [9] have clearly shown that the observed toxicity of commercial PS-NPs was induced by toxic water-soluble additives (e.g., antimicrobial NaN_3_ and surfactants) in the PS-NPs preparations, as revealed by the disappearance of toxicity after particle dialysis. Therefore, for studying the biological effects of NPs more and more, researchers produce and characterize their own NP material, and in the last several years, there has been an increasing number of publications reporting the preparation of NPs [3,8]. 

NPs are produced by either the “bottom-up” or “top-down” approaches. The “top-down” approach includes the laser ablation, photodegradation, ultra-sonication, or mechanical degradation of primary or secondary plastics. The disadvantages of this approach are irregular shape, surface defects, and oxidized surface, as well as the difficult and time-consuming preparation of large quantities of NPs [8]. The “bottom-up” approach stems from traditional colloidal chemistry methods such as conventional dispersed media (emulsions and nanoprecipitation), where ionic or non-ionic surfactants enable NPs dispersibility [8]. However, even after extensive NP rinsing, the residual traces of surfactants are difficult to remove [3]. The recent critical review highlighted the importance of the purification of PS-NP suspensions to remove additives, such as surfactants, before performing toxicity evaluations [10]. Because of the contribution of the surfactants to the observed toxicity, it remains critical to establish auxiliary chemical methods for the purity assessment of manufactured NPs, correlating toxicity studies with additional relevant parameters such as NP physicochemical characterization (size, shape, zeta potential) and other parameters related to the properties of the chemical type of plastic polymer and NP morphological characteristics.

In this work, we have synthesized NPs based on polyethylene terephthalate (PET) by the nanoprecipitation method using the ionic surfactant sodium dodecyl sulfate (SDS). We have established ^1^H NMR spectroscopy as a quality control for the presence of ionic surfactant throughout the purification procedure to monitor semi-quantitatively the ratio of surfactant to NPs. The modeled particles of the different stages of purification were subject to biological assays, such as red blood cell hemolysis, cytotoxicity, ROS production, apoptosis of primary mononuclear cells, and uptake by CD14+ primary mononuclear cells. We demonstrate that for a wide range of surfactant/NP ratios, no difference in the cytotoxicity was observed between the particles of different purification stages, but the trace amounts of surfactants present accounted for differences observed in protein-free media. 

## 2. Materials and Methods

### 2.1. Materials

#### 2.1.1. Materials for Preparation and Characterization of PET NPs

Polyethylene terephthalate (PET) (CAS: 25038-59-9, Cat. no. 429252) granules, as well as deuterated chloroform (>99% grade) were obtained from Sigma Aldrich-Merck (St. Louis, MO, USA). Bovine serum albumin (BSA) fraction V was purchased from Pan-Biotech (Aidenbach, Germany), while sodium dodecyl sulfate (SDS, analytical grade) was from Serva (Heidelberg, Germany). Trifluoroacetic acid (TFA, ≥99.5%, Optima™ LC/MS grade) was obtained from Fisher Chemical (Oxford, UK). LACE6C low speed centrifuge 3672× *g* (COLO LAbExperts, Novo Mesto, Slovenia) and ultrasonic bath (Sonorex Super RK52, 230 V, 50/60 Hz) from Bandelin electronic GmbH & Co. KG (Berlin, Germany) were used. All sonication steps were done by continuous sonication at 35 kHz and HF peak power 60 W. ^1^H NMR spectra were recorded with Varian/Agilent NMR 400 MHz. Deuterated chloroform (CDCl_3_) used for ^1^H NMR (>99.9%) was from Sigma Aldrich-Merck (St. Louis, MO, USA). Chemical shifts are expressed in ppm, while TMS (tetramethylsilane) was used as an internal standard. Milli-Q water (Smart2Pure 3 UV/UF, Thermo Scientific, Waltham, MA, USA) was used for all experiments.

#### 2.1.2. Materials for Cellular Assays

Human AB serum, penicillin, streptomycin, L-glutamate, 3-(4,5-dimethylthiazol-2-yl)-2,5-diphenyltetrazolium bromide (MTT), trypan blue dye, and dimethyl-sulfoxide (DMSO) were purchased from Sigma Aldrich-Merck (St. Louis, MO, USA). RPMI phenol red free medium was obtained from Life Technologies, Inc. (Rockville, MD, USA). Ficoll-Paque Plus gradient medium was from Cytiva (Uppsala, Sweden). Trypan blue dye was from Gibco (Thermo Fisher Sci, Waltham, MA, USA). Annexin V and propidium iodide (PI) were purchased from Becton Dickinson Bioscience (BD, Bioscience, San Jose, CA, USA). 2’,7’–dichlorofluorescin diacetate (DCFDA) cellular reactive oxygen species (ROS) assay kit was obtained from Abcam (Abcam, Cambridge, UK). The 20% solution of human serum albumin (HSA, 96% purity, intended for clinical use) was purchased from Baxter (Baxter, Vienna, Austria). All standard salts and glucose were purchased from Sigma Aldrich-Merck (St. Louis, MO, USA) in p.a. quality. Milli-Q water (Smart2Pure 3 UV/UF, Thermo Scientific, Waltham, MA, USA) was used for all experiments.

### 2.2. Methods

#### 2.2.1. Preparation of PET NPs

The model NPs used in this work were produced using the nanoprecipitation approach starting from commercial PET pellets. The pellets were used for the production of NPs without any shredding.

PET NPs were prepared according to the previously published precipitation protocol [11], with several modifications from [12,13]. The overall scheme of nPET production procedure is presented in Figure S1 (Supplementary Material). Briefly, 2.00 g of PET pellets were dissolved in 20.0 mL of 90% TFA in Milli-Q water (*v*/*v*), and stirred at 300 rpm for five hours at 50 °C s and then for 18 h at room temperature. In order to precipitate the NPs, 20.0 mL of 20% TFA in Milli-Q water (*v*/*v)* was added dropwise over 110 min to the original high-concentration TFA solution (1 drop of 10 μL per 3 s) under vigorous stirring at 1200 rpm using a dropping funnel. After completion of adding the TFA, stirring of the suspension was continued for an additional 2 h, before being sonicated in an ultrasonic bath for 15 min. Suspension was separated into 4 glass centrifuge tubes (each tube with about 10 mL of suspension) and centrifuged at 918× *g* for 40 min. After the supernatant was carefully removed using a glass pipette, Milli-Q water (10 mL) was added to each tube, sonicated for 10 s and centrifuged at 918× *g* for 40 min. After removing the supernatant, Milli-Q water (10 mL) was added to each tube, sonicated for 10 s, and centrifuged at 1632× *g* for 20 min. The washing of NPs with Milli-Q water was repeated until the pH of the suspension reached 6. 

The NP precipitates from 4 tubes were then combined and resuspended in 200 mL of 0.5% aqueous solution of sodium dodecyl sulfate (SDS) and ultrasonicated for 40 min. The resulting suspension was quickly transferred into a 250 mL cylinder and allowed to settle for 24 h. The top 100 mL containing a suspension of nanosized PET particles was separated and transferred into another cylinder. 

#### 2.2.2. NP Size Separation and Removal of Impurities

PET NP suspension in 100 mL of 0.5% SDS was carefully separated into nine fractions (each 10 mL), according to their sizes after particles settled in the presence of surfactant, starting from the top 10 mL of suspension in the cylinder (Figure S1), denoted NP-10, NP-20,… NP-90, up to 90 mL, and the last 10 mL of suspension was not used. Each suspension was transferred to glass centrifuge tubes for further purification from the largest particles and impurities like SDS (Figure S2). Fractions NP-10, NP-20, and NP-30 were used for preparation of PET NPs for further analysis and the assays on the cells.

In order to remove the largest particles, samples NP-10, NP-20, and NP-30 were firstly centrifuged at 102× *g* for 20 min (Figure S2) and the pellet was removed. After that, in order to remove the smallest particles, obtained supernatants were transferred to new tubes, and centrifuged at 1632× *g* for 20 min and supernatants were removed. The obtained pellets were resuspended in 10 mL of Milli-Q water using an ultrasonic bath for 10 s. One aliquot of this suspension (labeled as unwashed NPs in later text) was used for determination of zeta potential and cellular assays. The rest of the suspension was used for preparation of final NPs (labeled as washed NPs). In the first washing step, these suspensions were centrifuged at 1632× *g* for 40 min, and the supernatants were removed. Milli-Q water (10 mL) was added to pellets, sonicated for 10 s, and centrifugation was repeated under the same conditions. In order to remove soluble substances, such as surfactant SDS, monomer terephthalic acid (TPA), etc., the step of NP washing with Milli-Q water was repeated 5 times. Particles at different stages of purification were analyzed by ^1^H NMR and saved for cellular assays.

After 5 washes, NP-10, NP-20, and NP-30 pellets were combined and resuspended in 4.5 mL of Milli-Q water using an ultrasonic bath for 5 min. After that, the NP suspension was divided in three equal parts (3 × 1.5 mL). The first 1.5 mL of PET NPs suspension in Milli-Q water (labeled as washed NPs) was transferred to a glass vial, and used for determination of size distribution, zeta potential, and assays on cells. In order to exchange the medium in which NPs were suspended, the remaining two parts of NP suspension (1.5 mL each) were transferred into centrifuge tubes. After addition of Milli-Q water (8.5 mL), suspensions were centrifuged at 1632× *g* for 40 min. The supernatants were discharged and one pellet was suspended in 1.5 mL of 0.05% aqueous solution of BSA (NPs washed in BSA), while the other was suspended in 1.5 mL of 0.1% aqueous solution of SDS (NPs washed in SDS), using an ultrasonic bath for 5 min. Samples were stored at 4 °C until further use. 

#### 2.2.3. Preparation of NPs for NMR Analysis

^1^H NMR spectroscopy was used to check the presence of SDS in the prepared NPs, as well as for the monitoring of the washing process of NPs. 

The aliquot (1 mL) of NPs of different purification stages was first centrifuged at 1632× *g* for 40 min, and after removing the supernatant, the remaining water in the pellet was evaporated. The solid residue was dissolved in 0.5 mL of the TFA and deuterated chloroform (CDCl_3_) (*v*/*v*, 4:1) mixture. ^1^H NMR spectrum was recorded with 400 MHz NMR.

#### 2.2.4. Determination of Concentration of NPs in Water Suspensions

The concentration of NPs in water suspensions was determined gravimetrically [14]. Before the measurement of 0.3 mL suspension of NPs in Milli-Q water into previously weighed glass vials, the suspension was sonicated for 10 min. After measuring the mass of suspension on the analytical balance with an error of 0.1 mg, water was completely evaporated, and the remaining NPs were dried using high vacuum (2.25 mmHg) for 1 h. The concentration of NPs was calculated according to the following equation: concentration of NPs (mg/mL) = (mass of vial with dried NPs − mass of empty vial)/(mass of vial with suspension − mass of vial with dried NPs). 

#### 2.2.5. Determination of NP Size

The hydrodynamic diameter of the NPs was measured by dynamic light scattering (DLS) using the Malvern zetasizer Nano-ZS ZEN 3600 (Malvern Panalytical, Malvern, UK). One hour before the measurement, NP dispersions were held in an ultrasonic bath for 10 min. Measurement was performed with a 60 s equilibration period at 25 °C in triplicate without moving the cuvette (runs of 1 measurement: 10; run duration: 10 s; delay between runs: 0 s). Parameters for size calculation: 173° backscatter detection; material RI: 1.636; dispersant: water; dispersant RI: 1.330; viscosity (cP): 0.887.

#### 2.2.6. Determination of Zeta Potential of NPs 

Zeta potentials of NPs at different stages of purification from the surfactant were measured in Milli-Q water or 0.5% SDS using the Malvern zetasizer Nano-ZS ZEN 3600 according to [15], in a DTS1070 cuvette (Malvern Panalytical, Malvern, UK). For measurement, the same parameters were used as for the determination of NP size.

### 2.3. Effects of NPs on Cells

#### 2.3.1. Isolation of Peripheral Blood Mononuclear Cells (PBMCs)

Buffy coats were obtained at the Department of Transfusion Medicine at Karolinska University Hospital and diluted 1:1 with phosphate-buffered saline (PBS). The cell solution was carefully layered on top of Ficoll-Paque Plus in 50 mL PP tubes and PBMCs were separated by 400× *g*, 30 min centrifugation without brake. The interphase was carefully collected and washed twice with PBS at 300× *g*, 10 min. PBMCs were counted using trypan blue in a Bürker counting chamber.

For all experiments, PBMCs were incubated with NPs in cRPMI medium (complemented with 2% heat-inactivated human AB serum, L-Glutamine, penicillin, and streptomycin) was used.

#### 2.3.2. MTT Assay

The MTT assay was done according to Mosmann [16], with modifications. PBMCs were seeded (150,000 cells per well in a final volume of 150 µL) in 96-well plates stimulated with different NPs (washed or unwashed) in a concentration range (0.001 to 100 µg/mL). The untreated control represented cells without stimulant, while positive control were cells pretreated with 33% DMSO. After 24 h incubation at 37 °C in a humidified atmosphere with 5% CO_2_, a solution of MTT was added to each well and mixed to allow for the metabolization of MTT. After incubation for 4 h, the medium was removed by centrifugation for 5 min at 300× *g* and aspiration by pipette tips, and formazan crystals were resuspended in 100 μL DMSO. The absorbance was read at 570 nm. Every stimulus was tested in duplicates (two wells).

#### 2.3.3. Annexin V Assay

The induction of apoptosis by NPs of PBMCs was determined by a flow cytometry methodology by van Engeland et al. [17], using Annexin V-FITC and propidium iodide (PI), according to the manufacturer’s instructions (BD Bioscience, San Jose, CA, USA). Briefly, 1 × 10^6^ cells/mL in 200 µL of cell medium in FACs tubes (crystal clear polystyrene tubes, dimensions 12 × 75 mm), treated with various concentrations of washed and unwashed NPs (0.1–100 µg/mL) for 24 h, were harvested and washed out with PBS. After, cells were resuspended in a binding buffer containing Annexin V-FITC and PI, incubated for 20 min, and immediately after analyzed by flow cytometry using a FACSCanto II cytometer (BD Bioscience, San Jose, CA, USA) with at least 20,000 events recorded. The FlowJo v10 software (TreeStar Inc., Ashland, OR, USA) was used to analyze the data. Controls were unstained cells, cells stained only with Annexin V, and cells stained only with PI. 

#### 2.3.4. DCFDA Cellular ROS Assay

For induction of ROS, the DCFDA assay was performed according to Calamita et al. [18], with washed and unwashed NPs in different concentrations (0.1–100 μg/mL), 1 × 10^6^ cells/mL in 500 μL of cell medium in FACs tubes (crystal clear polystyrene tubes dimension 12 × 75 mm) were stimulated for 4 and 24 h at 37 °C in a humidified atmosphere with 5% CO_2_. Cells were washed with PBS, and half of the volume was stained with 5 μM H_2_DCFDA in PBS for 1 h at 37 °C. The other half of the cells were used for the uptake experiment (see below). Samples were analyzed by flow cytometry as stated above, where at least 50,000 events per sample were recorded. Positive control for ROS induction was 10 mM H_2_O_2_, added to the cells 1 h before staining. For proper gating, non-stained cells were used. Induction of ROS was analyzed based on fold change from unstimulated non-stained cells. The experiment was performed in two independent cell donors.

#### 2.3.5. Uptake of NPs by Monocytes

Isolated PBMCs (250,000 cells) were stimulated with different washed and unwashed NPs, as stated above. After 4 and 24 h of stimulation, cells were washed with PBS and stained with anti-CD14 antibodies in APC or APCH7 (BD Bioscience, San Jose, CA, USA) for 30 min at 4 °C. After the washing step, cells were analyzed by flow cytometry, where at least 50,000 events per sample were collected. CD14 gating was based on unstained cells. The uptake of NPs was analyzed in FlowJo v 10, looking at the MFI of the size scatter channel. The experiment was performed in two independent cell donors.

#### 2.3.6. Hemolysis of Red Blood Cells

##### Preparation of Red Blood Cells (RBCs) Suspension

Human blood was obtained from healthy donors using an EDTA vacutainer and 21-gauge needle (BD Vacutainer^®^, Franklin Lakes, NJ, USA) on the day of the experiment. The plasma and buffy coat were removed after centrifugation for 5 min at 3890× *g* (Eppendorf^®^ Minispin^®^, Hamburg, Germany). RBCs were washed once with PBS, twice with 0.99% NaCl, and once with Ringer’s solution (NaCl 8.6 g/L, KCl 0.3 g/L, CaCl_2_ 0.33 g/L, NaHCO_3_ 0.2 g/L, pH 7.4). Isolated RBCs (0.150 mL) were resuspended in 50 mL of 0.99% NaCl or Ringer’s solution with or without 5 mM glucose and 45 g/L HSA.

##### Preparation of Incubation Mixture of RBC and NPs and Measurement of RBCs Hemolysis

The hemolytic effect of NPs on RBCs was measured in supernatant at 540 nm, using Shimadzu UV/VIS 1800 (Kyoto, Japan), after incubation of the prepared mixtures of RBCs and NPs for 3 h at room temperature and centrifugation for 2 min at 6810× *g* (Eppendorf^®^ Minispin^®^, Hamburg, Germany). Incubation mixtures consisted of 0.9 mL RBCs suspended in 0.99% NaCl or Ringer’s solution with or without 5 mM glucose and 45 g/L has and 0.1 mL suspension of PET NPs in Milli-Q water. Washed PET NPs and unwashed PET NPs were used for the preparation of suspensions in Milli-Q water in the concentrations of 1, 10, and 100 μg/mL. Final concentrations of NaCl and washed and unwashed PET NPs in the incubation mixture were 0.9% NaCl, and PET NPs concentrations were 0.1, 1 and 10 μg/mL, respectively. The control mixtures were prepared with 0.9 mL RBCs suspended in 0.99% NaCl and 0.1 mL Milli-Q water (negative control), 0.9 mL RBCs suspended in 0.9% NaCl and 0.1 mL 0.9% NaCl (negative control), 0.9 mL RBCs suspended in 0.99% NaCl and 0.1 mL 0.1, 0.03 and 0.003% SDS in Milli-Q water (surfactant controls), or 0.9 mL RBCs in Ringer’s solution (with or without 5 mM glucose, 45 g/L HSA) and 0.1 mL Milli-Q water. RBCs (0.9 mL in 0.9% NaCl) lysed with 1% Triton-X 100 (0.1 mL) were used as a positive control (100% hemolysis). All incubation mixtures were set up in triplicate. The percentage of hemolysis was calculated according to the following equation: percentage of hemolysis (%) = 100 × A540 of sample/A540 of 1% Triton X-100.

### 2.4. Statistics and Graph Generation

Each assay was, if not stated otherwise, performed in duplicate. The results are expressed as mean ± standard deviation (S.D.). The statistical significance of obtained differences between samples were tested using a two-sample *t*-test and ANOVA one-way test with Tukey’s multiple comparison test. All statistical analysis and graphical representations of data were performed using the GraphPad Prism statistical program. *p* values less than 0.05 were considered as significant.

### 2.5. Ethics

In this study, buffy coats were bought from Karolinska University Hospital, where ethical permits are not required. Experiments with human blood were approved by The Swedish Ethics Review authority (Etikprövningsmyndigheten) with permit number No. 20112085-31/4. The study was performed following the declaration of Helsinki.

## 3. Results

### 3.1. PET NPs Characterization

#### 3.1.1. Yield and Concentration Determination of Synthetized NPs

Using a gravimetric method, the yield and concentration of the synthetized NPs were estimated. For the combined NP suspensions, the average concentration of 1.13 mg/mL was determined by the gravimetric method, giving an overall yield of 0.76%. Three NP batches are fractionated according to size (from fractions NP-10 to NP-30, NP-40 to NP-60, NP-70 to NP-90) giving a yield of 0.97%, 0.73%, and 0.58%, respectively. 

#### 3.1.2. PET NP Size Distribution

According to the DLS measurements, the synthetized washed PET NPs, dispersed in Milli-Q water, BSA (0.05%), and SDS (0.1%), as well as the unwashed NPs, showed an average size distribution in the 200–300 nm range, with a relatively wide polydispersity index (around 0.4) (Table 1, Figures S3 and S4). Moreover, a similar average size distribution was measured also for the PET NPs prepared from the fractions NP-40 to NP-90 (Table S1 and Figures S5–S10). The hydrodynamic average distribution of NPs washed and dispersed in water is just slightly higher than in the presence of SDS and BSA, suggesting that even when the NPs are dispersed in the absence of surfactants, there is no NP agglomeration.

#### 3.1.3. Determination of SDS Level in Corona of PET NPs by ^1^H NMR

**Figure 1:**
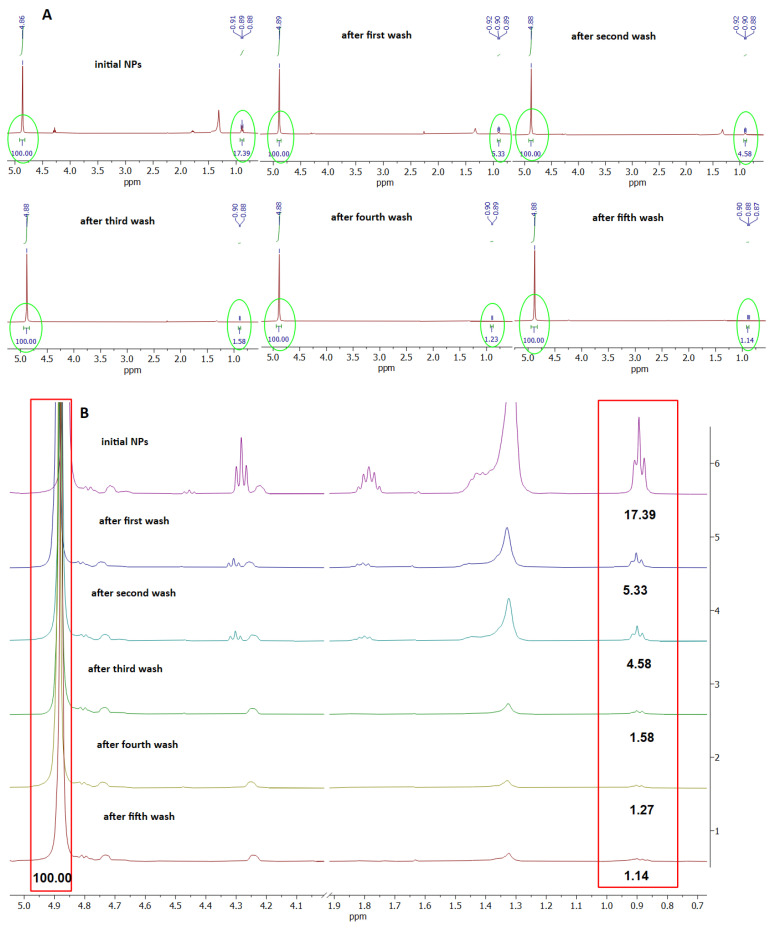
^1^H NMR spectra of PET NPs preparation before and after washing steps. (**A**). ^1^H NMR spectra in region 5.0 to 0.6 ppm; (**B**). Zoomed in view of characteristic chemical shifts of PET and SDS protons, taken for relative quantification of SDS present in PET NPs. Relative ratio of SDS: PET is in the red box in the figure.

As no particle aggregation was observed when the NPs were dispersed in Milli-Q water, we supposed that the SDS remaining in the NP coronas after the extensive washing of the NPs could be responsible for their dispersibility. Therefore, we intended to compare the level of SDS in the corona of the NP preparation, before and after every washing step, using ^1^H NMR (Figure 1A,B). Figure 1A presents the ^1^H NMR spectra of the NP preparation before and during all the washing steps in the region 5.0 to 0.7 ppm. Figure 1B presents a zoomed out spectra in the region 0.7 to 1.9 ppm and 4.0 to 5.0 ppm. The peaks at 0.9, 1.35, 1.81, and 4.3 ppm originate from the SDS, while the peaks at 4.88, 4.75, and 4.26 ppm are from the PET (Figure 1B).

The level of SDS in all samples was estimated based on the ratio of the characteristic peak of the terminal methyl group of SDS (signal multiplicity is triplet) centered at 0.9 ppm (0.88–0.92 ppm) and the peak of the internal methylene groups of PET (signal multiplicity is singlet) centered at 4.88 ppm. It can be observed that the ratio of SDS/PET gradually decreases from 17.39 to 1.14 during extensive washing with water. However, this ratio reaches a plateau already after the third wash (ratio 1.58), with only a negligible decrease after two further washings (Figure 1B and Figure S11), finally decreasing the level of SDS in the NP coronas by 15.25 times. Since during every washing step, about 200 μL of NPs were washed with 10 mL of water, it is obvious that a certain amount of SDS was adsorbed at the NP surface, with a low constant of desorption, thus resisting complete removal. These results suggest that the adsorbed SDS forms a negatively charged surface on the NPs, resulting in NP repulsion, explaining the absence of NP aggregation in water. The ^1^H NMR spectra of the solvent mixture, starting material (PET pellet), and SDS are presented in Figures S12–S14.

#### 3.1.4. Zeta Potential Determination of PET NPs

As the SDS remained adsorbed in the NP coronas even after their extensive washing, the zeta potential of several NP preparations was compared (Table 2). The NPs washed and dispersed in Milli-Q water have a zeta potential of −34.93 mV, demonstrating a profound negative charge on the NPs’ surface, pointing to the presence of adsorbed SDS. When the washed NP preparation was dispersed in 0.5% SDS, the zeta potential is profoundly lower (−63.53 mV), suggesting that additional SDS molecules from a dispersant were adsorbed on the NPs’ surface, making its charge more negative. The unwashed NP preparation had a zeta potential of −42.10 mV. 

An increase in the zeta potential after repeated NP rinsing was also reported for the poly(methyl methacrylate) and poly(vinyl chloride) NPs produced by precipitation in the presence of SDS [19]. The zeta potential of the PET NPs obtained by milling and dispersing in 0.1% SDS was −66.7 mV, and in 1000 times diluted SDS (0.0001% SDS) increased to −23.2 mV [12]. Aguilar-Guzmán et al. [20] produced PET NPs by nanoprecipitation and observed a zeta potential of −47.5 mV for the PET NPs of a hydrodynamic radius of 221 nm in 0.5% SDS, while after washing twice with ultrapure water and absolute ethanol and resuspension in water, the zeta potential was +3.6 mV, with a hydrodynamic radius of 2660 nm. This suggests that if SDS is completely removed, insufficient charge on the NPs’ surface results in NP agglomeration. Interestingly, although Bashirova et al. [21] did not use surfactants during PET NP preparation by the precipitation method, their reported zeta potential was −20 ± 5 mV. Similarly, PET NPs prepared by laser ablation also without the involvement of surfactants had a zeta potential of −43 mV [22]. Therefore, it could not be excluded that in our preparation of washed NPs, the adsorbed SDS contributes to the low value of the zeta potential measured in the final preparation. 

### 3.2. Effects of Residual Ionic Surfactant in NP Preparations in Cellular Assays

#### 3.2.1. Cytotoxic Effects of PET NPs on Human Peripheral Blood Mononuclear Cells (PBMCs)

**Figure 2:**
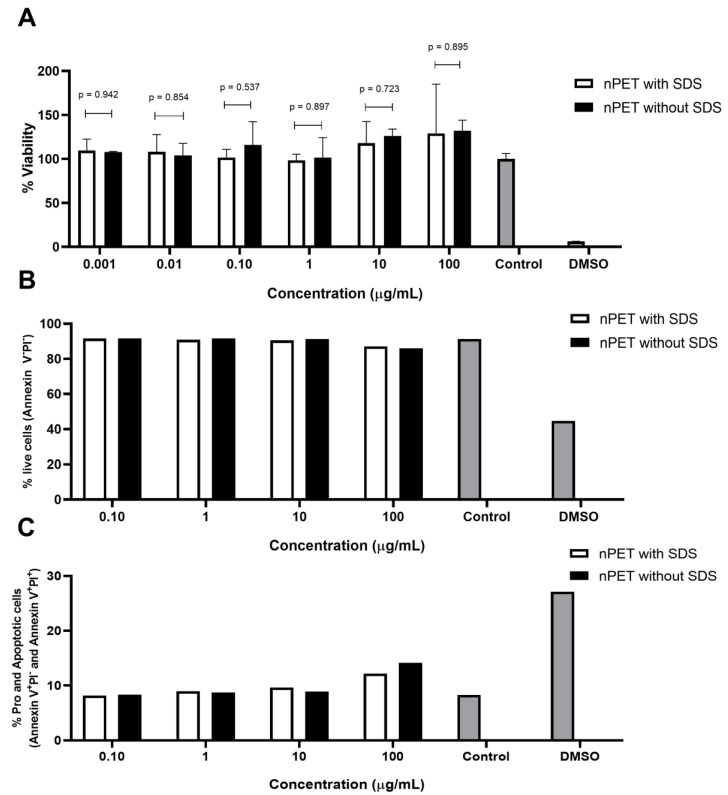
Cytotoxicity of PET NPs on human PBMCs tested by MTT (**A**) and apoptosis (**B**,**C**) assays obtained after PBMCs were exposed to PET NPs of different purities for 24 h, nPET with SDS (unwashed), and nPET without SDS (washed). Data from apoptosis assay are representative data from one PBMC donor.

The presence of impurities, such as the presence of surfactants (e.g., cetyltrimethyl ammonium bromide or hexadecyl trimethylammonium chloride used to make mesoporous silica) may influence the results of toxicity evaluations [23]. The NP preparations of high purity with a low amount of surfactant (washed PET NPs) and an intermediate level of purification (unwashed PET NP) were subject to cytotoxicity testing by the MTT assay on peripheral blood mononuclear cells (PBMCs). No statistically significant acute (24 h) toxicity was observed in either preparation of the NPs for all the tested concentrations of NPs (Figure 2A, *p* > 0.05), suggesting that the NPs do not exert acute toxicity. These results also demonstrate that NPs with either surfactant/NP ratio, 17.39 in unwashed NPs and 1.14 in washed NPs, do not have toxic effects. In the study of Magrì et al. [22], no alterations in the cell viability were observed after Caco-2 cell treatment nor with PET NPs (136 nm) at 30 μg/mL even for 96 h, neither with PET NP–contaminant complexes (glyphosate, levofloxacin, and Hg^2+^ ions), suggesting a lack of short-term negative effects in the cells. By contrast, an apparent increase in the % viability was noted in higher concentrations of PET NPs applied in the test, similarly to a previously observed effect in a macrophage cell line (50–250 nm) [20], and human lung carcinoma A549 cells (164 nm) [13]. An increase in the cell viability was also observed in the presence of PS NPs (100 nm) in gastric adenocarcinoma cells at 10 μg/mL [24].

Several studies observed no inhibitory effects of secondary PET NPs in a similar concentration range (1–100 μg/mL) in different cell lines, such as primary human nasal epithelial cells (NPs 100 nm) [25] or the human lymphoblastic cell lines THP1 and TK6 (NPs 100 nm) [15]. An inhibitory effect of the primary PET NPs (164 nm) on the A549 cell viability was observed at concentrations of 100 and 200 μg/mL [13].

In addition to the PET NPs, also, NPs of other types (such as PS) and lower sizes (50 nm) have no acute toxic effect at concentrations < 100 μg/mL. For the PS NPs (50 nm), the effects on the cell viability of the peripheral blood lymphocytes were obtained only at very high concentrations: 73.1% and 39% viability at 500 μg/mL and 2000 μg/mL, respectively [26]. No effect on the Caco-2 cell viability was obtained with the PS NPs (50 nm) at 39 μg/cm^2^ [27] and up to 240 μg/mL [28]. With 24 h of exposure, no pronounced loss of human umbilical vein endothelial cell viability was observed in the presence of 100 nm or 500 nm PS-NPs, even when the PS-NP concentration was up to 100 μg/mL [29]. Therefore, regardless of the NP type and size, as well as the type of cell (primary or secondary cell line of different types), in general, NPs have cytotoxic effects only at high concentrations (>100 μg/mL). The main reason for this could be the protective effect of the media, e.g., the binding of the NPs to media components, mainly proteins. Mahadevan et al. [19] demonstrated that even at a concentration of 0.4%, SDS was nontoxic to the BHK-21 cells, while 4% SDS decreased the cell viability only by 30%, and this seems to be also due to the protective effect of the protein-rich cell culture medium.

**Figure 3:**
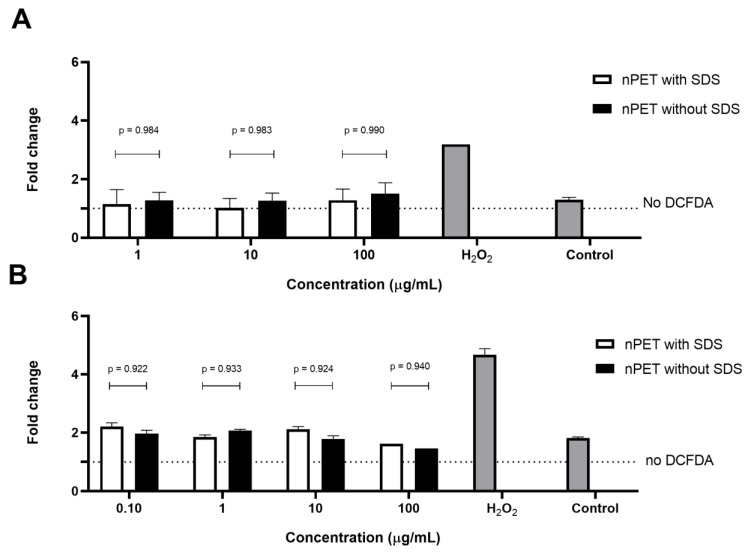
ROS production in human PBMCs was monitored after 4 h (**A**) and 24 h (**B**) of exposure to PET NPs of different purities, nPET with SDS (unwashed), and nPET without SDS (washed).

The results of the apoptotic measurements (Figure 2B,C) and ROS determination (Figure 3) in human PBMCs exposed to NPs were in line with the low cytotoxicity of the NPs observed in the MTT assay (Figure 2A). The percentage of live cells, and pro- and apoptotic cells was analyzed by the determination of PI incorporation using flow cytometry. Only the highest concentration (100 μg/mL) of NPs applied showed limited effects on cell apoptosis induction and ROS generation, but it was not statistically significant (*p* = 0.99). In accordance with the cytotoxicity, the NPs with either surfactant/NP ratio, 17.39 in unwashed NPs and 1.14 in washed NPs, did not increase the ROS generation, nor induce apoptosis (Figure 2B,C and Figure 3A,B). Similarly, Annangi et al. [25] have found a significant increase in ROS when primary human nasal epithelial cells were exposed to 100 µg/mL PET NPs (100 nm). Aguilar-Guzmán et al. [20] reported that, in spite of PET NPs inducing an increase in the % of viability, PET NPs induced a slight but significantly increased intracellular ROS production at all concentrations assayed, although this ROS increase did not correlate to the PET-NP concentration increment. Zhang et al. [13] also have found that PET NPs induced ROS increase at a concentration of 50 μg/mL, but without the induction of cell apoptosis. However, as PBMCs are resistant to oxidative stress, similarly to macrophages [30], slightly increased ROS production at the highest NP concentration tested in our study is probably not deleterious to them, resulting in an observed lack of cytotoxic effects. Domenech et al. [27] also found that PS NPs (up to 6.5 μg/cm^2^) did not increase ROS in Caco-2 cells after 24 h, and even after 8 weeks of exposure. 

#### 3.2.2. Hemolytic Effects of PET NPs on Human Red Blood Cells 

It has previously been observed that NPs can interact with cell membranes. Severe effects during the process of the biological interactions of NPs and cell membranes include damage of the membrane structure and cell death. Various factors, such as type and surface charge, play an important role in this process. For example, polyethylene NPs fuse with the hydrophobic core of lipid bilayers and further form a network of disentangled, single polymeric chains. These complexes promote damage to the membrane structure and fluidity, and ultimately, cell death [31]. Red blood cells (RBCs) are particularly sensitive to membrane damage and burst if affected, releasing hemoglobin into solution that can easily be monitored by measuring the absorbance at 540 nm to estimate the magnitude of hemolysis. Therefore, the hemolytic effect of the washed and unwashed PET NPs was determined using RBCs obtained from three healthy donors. 

**Figure 4:**
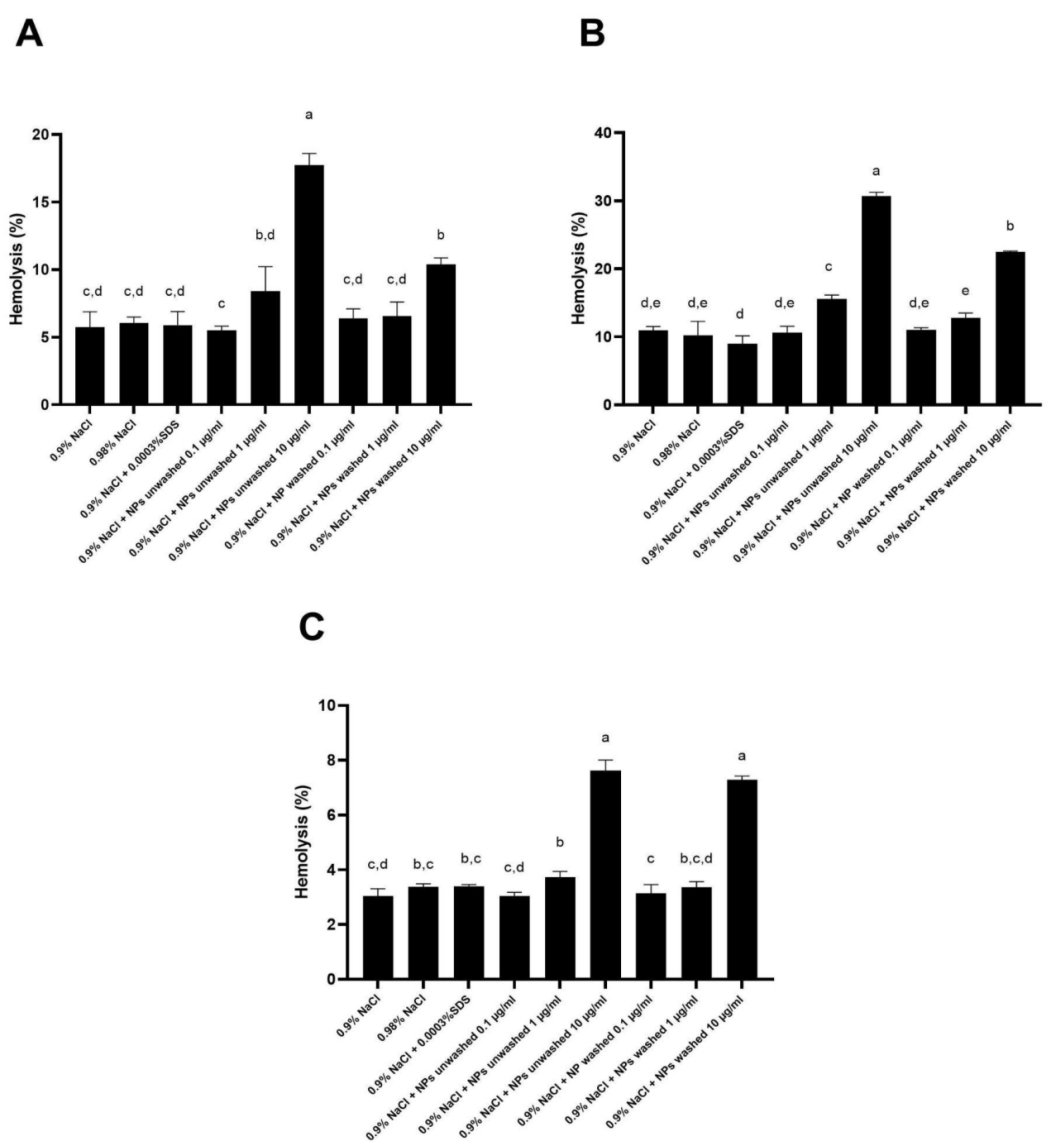
Percentage of hemolysis in the presence of washed PET NPs and unwashed NPs (with SDS) determined by incubation of human red blood cells (RBCs) from three healthy donors ((**A**)—donor No. 1, (**B**)—donor No. 2 and (**C**)—donor No. 3) in 0.9% NaCl. The final incubation mixture consisted of 0.9 mL RBCs in 0.99% NaCl and 0.1 mL of tested solutions: water, 0.9% NaCl, 0.003% SDS, unwashed PET NPs at 1, 10, and 100 μg/mL and washed PET NPs at 1, 10 and 100 μg/mL. Final concentrations of NaCl and washed and unwashed PET NPs in the incubation mixture are 0.9% NaCl, and PET NPs at 0.1, 1, and 10 μg/mL, respectively. The percentage of RBC hemolysis was calculated in relation to the hemolysis obtained for 1% Triton X-100 (100% hemolysis). Data were analyzed using one-way ANOVA with Tukey’s multiple comparison test at a significance level of 0.05. Statistical significant differences between samples were denoted a–e.

Depending on the RBC donor, the presence of SDS in the NP preparation (washed and unwashed) and the tested concentration of NPs, the obtained percentages of hemolysis were between 3 and 33% in comparison to 1% Triton X-100 (100% hemolysis (Figure 4)). Similarly, for the amine-modified PS NPs (100 nm) at 50 μg/mL and 100 μg/mL, human RBCs hemolysis at 10% and 26% was observed when incubated in Tris-buffered saline [32]. Interestingly, RBCs exposed to 100 µg/mL of 100 nm sized plain or carboxyl-modified PS NPs did not cause the hemolysis of human RBC [32].

**Figure 5:**
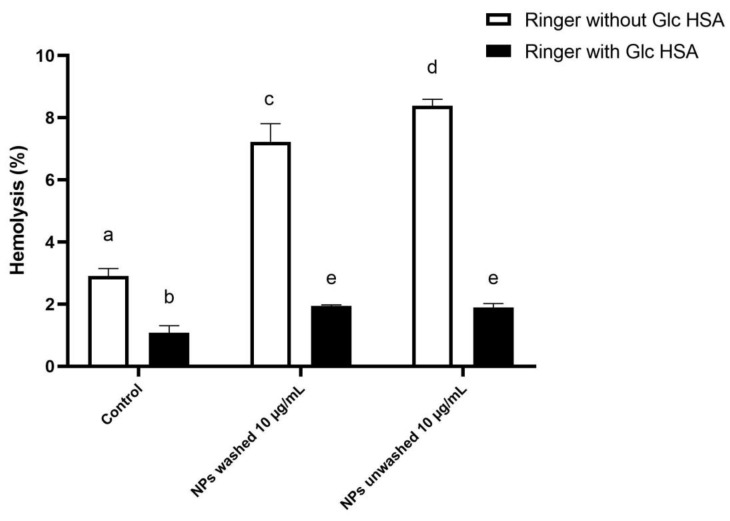
Percentage of hemolysis in the presence of PET NPs washed and NPs unwashed (with SDS). determined by incubation of human red blood cells (RBCs) from one healthy donor (donor 1) in Ringer’s solution with glucose and HSA. The final incubation mixture consists of 0.9 mL RBCs in Ringer’s solution with or without 5 mM glucose and 45 g/L HSA and 0.1 mL of tested solutions: water, washed PET NPs 100 μg/mL, and unwashed PET NPs 100 μg/mL. Final concentrations of NaCl and washed and unwashed PET NPs in the incubation mixture are 0.9% NaCl, and PET NPs 10 μg/mL, respectively. The percentage of RBC hemolysis was calculated in relation to the hemolysis obtained for 1% Triton X-100 (100% hemolysis). Data were analyzed using one-way ANOVA with Tukey’s multiple comparison test at a significance level of 0.05. Statistical significant differences between samples were denoted a–e.

For all three donors, a statistically significant (*p* < 0.05) increase in the hemolysis of the RBCs was obtained with 1 µg/mL of the unwashed sample of NPs (designated unwashed NPs 1 µg/mL), but not for the washed sample of the NPs (designated washed NPs 1 µg/mL) for the same concentration. Both the washed and unwashed NPs at the concentration of 10 µg/mL caused a statistically significant increase in the hemolysis of RBC. A statistically significant (*p* < 0.05) difference in the hemolysis of RBCs between the PET NPs without and with SDS was obtained for a concentration of 100 µg/mL for two out of the three donors (donors 1 and 2 in Figure 5).

According to these results, it could be concluded that an increase in the RBC hemolysis was caused by increasing the concentration of the NPs from 1 to 10 µg/mL and the presence of bound SDS in the soft corona. The more evident effect of the bound SDS in the soft corona of the NPs on the hemolysis of the RBCs was noticed in the unwashed NP sample for donors 1 and 2 compared to the washed NP sample. The different ranges of the hemolysis percentages obtained for the highest concentration of the NPs were expected because of the individual differences between the blood donors like the count of the RBCs, content of hemoglobin, and vulnerability of the RBCs to hemolysis caused by lifestyle (diet, smoking, alcohol consumption). Besides, when the effect of the SDS in the concentrations 0.0003, 0.03, and 0.01% on the percentages of RBC hemolysis was tested, it was found that the lowest concentration of SDS had an effect like that of Milli-Q water and 0.9% NaCl, while 0.03% and 0.1% SDS caused 100% hemolysis (results are not shown), suggesting that there is quite a narrow range of SDS concentrations to which cells respond with 100% hemolysis (10×). Therefore, a 15-fold time difference in the amount of SDS adsorbed to the NPs clearly caused a statistically significant difference observed in the response of the RBCs between the different NP preparations (washed vs. unwashed) and for the unwashed NPs, decreased the concentration threshold to which the cells responded with lysis. 

To check for the possible protective effects of substances present in plasma on the RBC hemolysis by the NPs, in the second experiment, the RBCs were incubated in Ringer’s solution, which contains 5 mM glucose and 45 g/L HSA (Figure 5). It was found that Ringer’s solution with glucose and HSA at the physiological concentrations significantly (*p* < 0.05) reduced the hemolysis of the RBCs, compared to the hemolysis obtained in Ringer’s solution for both samples of the washed and unwashed NPs, as well as in the negative control (Milli-Q water). There was no significant (*p* < 0.05) difference obtained for the unwashed and washed NPs samples in Ringer’s solution with glucose and HSA. Moreover, the obtained hemolysis for both NPs in the presence of glucose and HSA was significantly (*p* < 0.05) lower compared to the hemolysis obtained in the negative control prepared with Ringer’s solution. The obtained results confirm the observation of Barshtein et al. [33], that human RBCs hemolysis by PS NPs (500 μg/mL) is inhibited by the supplementation of the suspension medium (PBS) with albumin even at very low concentrations (0.05% wt). This suggests that proteins present in the incubation mixture in physiological concentrations can prevent the toxic effects of NPs on RBCs, e.g., that the presence of protein molecules on the NPs’ surface strongly modulates their interaction with RBCs and/or stabilizes the membrane. Therefore, in the assays performed in the cell culture of the primary mononuclear cells, where the NPs were added to the cell culture medium, the formation of the protein corona on the NPs modulates their effects on cells in a similar, most likely in a protective, manner (Figure 2 and Figure 3). These results can explain why no acute (24 h) toxic effects of the different types and sizes of the plastics on the different cell lines generally were not observed at the concentration of NPs up to 100 μg/mL, for the PET NPs [13,15,20,22,25], nor for the PS NPs [26,27,28,29]. 

According to the results of this study, as well as the results of other researchers, it seems that besides the standardization of the type, size, and concentration of the NPs used for biological assays, the standardization of protocols in the biological assays are also needed if we want to understand the realistic toxic effects of NPs in biological systems.

On the other hand, Kim et al. [32] demonstrated the uptake of amine-PS NPs into human RBCs and the morphological and functional changes in RBCs even at non-hemolytic concentrations, with the externalization of phosphatidylserine, generation of microvesicles in RBCs, and perturbations in the intracellular microenvironment. This suggests that internalization and morphological/functional changes precede hemolysis, and that the presence of SDS in our NPs may favor these changes, finally resulting in hemolysis.

#### 3.2.3. Uptake of NPs by CD14+ Monocytes

**Figure 6:**
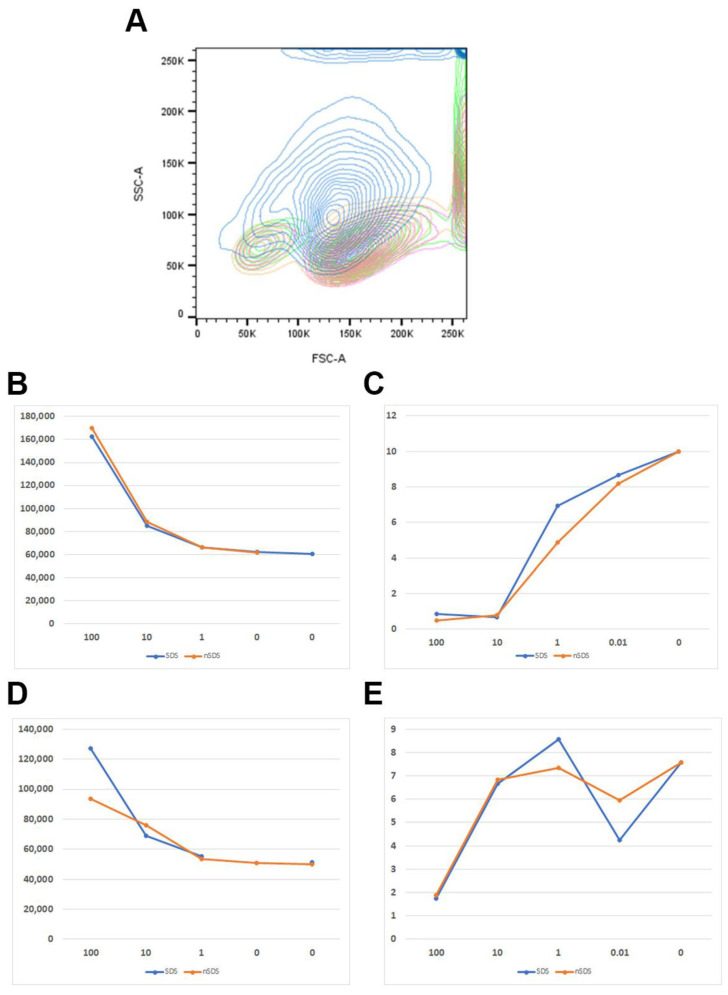
(**A**). Example of side scatter shift observed for CD14+ cells of human PBMCs in the presence of different concentrations of NPs (blue—100 μg/mL, orange—10 μg/mL, pink—1 μg/mL, green—control). (**B**). PET NP concentration-dependent side scatter shift of CD14+ cells of PBMC (unwashed SDS-NPs; washed SDS-NPs) for donor 1. (**C**). Percentage (%) of CD14+ cells in PBMC in the presence of different concentrations of PET NPs of different purity (unwashed SDS-NPs; washed SDS-NPs) for donor 1. (**D**). PET NP concentration-dependent side scatter shift of CD14+ cells of PBMC (unwashed SDS-NPs; washed SDS-NPs) for donor 2. (**E**). Percentage (%) of CD14+ cells in PBMC in the presence of different concentrations of PET NPs of different purity (unwashed SDS-NPs; washed SDS-NPs) for donor 2.

**Figure 7:**
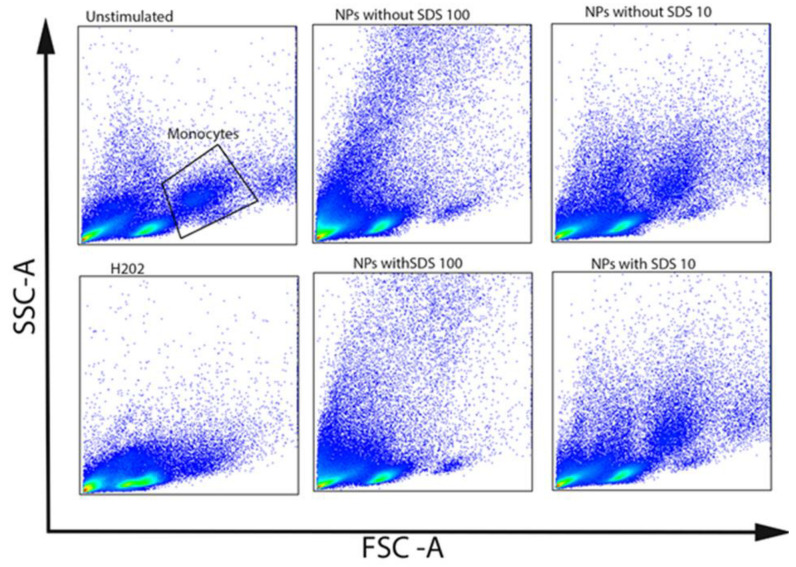
Forward and side scatter plots of PBMCs incubated with NPs with and without SDS at the concentrations of 100 and 10 μg/mL.

Both the in vivo and in vitro experiments indicate that NPs can penetrate cell membranes and can be internalized into cells, inducing intracellular biological effects. It has been shown that the cellular granularity (measured by flow cytometry side scatter) can be predictive of NP accumulation in cells [34,35]. The PET NPs of different stages of purity were tested for uptake by the CD14+ monocytes of human PBMCs. The side scatter shift, as a measure of uptake, was monitored for the different concentrations of the NPs, for which the two donors of PBMC were analyzed (Figure 6 and Figure 7). The NPs were uptaken in a concentration-dependent manner. A high side scatter shift was observed for the highest concentration tested, but it can also be seen in the low NP concentration (Figure 6B,D). Concomitantly, the % of CD14+ cells dropped down with the increase in uptake (Figure 6C,E), suggesting that apoptotic processes observed in PBMC may preferentially affect the population of antigen-presenting cells and CD14+ monocytes (Figure 6C,E and Figure 7). Differences between the NPs of different stages of purity with regards to the surfactant contents could not be observed. However, the CD14+ monocytes appeared to be more sensitive than the other PBMCs (Figure 7) and could not be protected by the protein-rich cell medium. There were also donor-specific differences in the CD14+ cells’ sensitivity to the NPs, but not with regards to the presence of the surfactant (Figure 6C,E).

Magrì et al. [22] showed that PET NPs (100 μg/mL) were mostly internalized in the endo-lysosomes of human Caco-2 intestinal epithelial cells. Similarly, Annangi et al. [25] observed PET NP (100 μg/mL) internalization in primary human nasal epithelial cells, supposing their localization in endosomes. In the study of Johnson et al. [14], mouse macrophage cells (RAW 264.7) were exposed to PET NPs (50 μg/mL). NPs were observed inside phagocytic bodies, where several cells had formed a tight phagosome around the NPs. Aguilar-Guzmán et al. [20] observed the internalization of PET NPs (15 μg/mL) by RAW 264.7 and revealed the mechanism of internalization: macrophages recognize PET NPs as a foreign particle, internalize it by macropinocytosis, and once inside, the small NP cluster is separated into individual vesicles (phagosome). A similar mechanism could be expected for the CD14+ monocytes of PBMC, thus explaining the NPs’ concentration-dependent increase in the number of individual phagosomes within cells, as reflected in the observed increased cell granularity. 

In vivo, antigen-presenting cells most likely uptake very low quantities of NPs, but if NPs are not degraded, chronic uptake can result in heavily loaded cells showing a number of disturbances that may impact their function, as demonstrated for PS NPs in mouse macrophages [30]. The absence of measurable cytotoxicity does not mean that NPs have no effect on cells. A recent study [36] observed extensive proteome changes in the absence of the toxic effects of PS NPs on the J774A.1 cell line (mouse macrophages), which are mostly adaptive changes, and proposed that NPs may affect the fine functioning of the innate immune system and have indirect adverse effects. Therefore, although no significant difference in the PBMC response to unwashed NPs and washed NPs was observed in our study, it cannot be excluded that the presence of SDS induced a different set of adaptive changes in cell proteome, particularly in chronic exposure settings. Ionic surfactants, as well as other cell metabolism active impurities introduced during the NP production should be carefully monitored and reported to allow for the meaningful comparison of the biological effects of NPs observed in different studies.

## 4. Conclusions

In this study, we have shown that nanoprecipitated PET produced from PET granules of high purity, binds ionic surfactant, needed for NPs dispersion, in its corona throughout the purification procedure. In cellular assays sensitive to ionic surfactants (such as the hemolysis of red blood cells) and particularly in the absence of proteins that may mitigate the negative effects, the presence of SDS lowers the threshold of the cellular response to NPs and accounts for the statistically significant differences observed between the NPs of different purity levels. We have shown that the relative ratio of surfactant (SDS) to PET NPs could be monitored by ^1^H NMR on the basis of the chemical shift characteristics of PET and SDS. As the size, shape, and zeta potential of NPs may all be relevant parameters for the biological effects of NPs, standardization in NP production methods and the quality control of manufactured particles with auxiliary chemical methods that track production impurities is of utmost importance for the ongoing risk assessment of NPs to enable the comparison of the results obtained in different in vitro and in vivo studies. Our study strongly supports the recent review of the summarized knowledge of the toxicity of nanoplastics to aquatic organisms, where a separate section was dedicated to problems in the study of NP toxicity due to the presence of NP impurities [2]. 

## Figures and Tables

**Table 1:** Size distribution (d.nm) and polydispersity index (PdI) of PET NPs washed and dispersed in Milli-Q water, BSA (0.05%), and SDS (0.1%), and NPs unwashed and dispersed in Milli-Q water determined by DLS.

Samples	Dispersant	Z-Average (d.nm)	Polydispersity Index (PdI)
NPs washed	Milli-Q water	311.7 ± 4.4	0.375 ± 0.026
	BSA (0.05%)	243.5 ± 1.3	0.352 ± 0.037
	SDS (0.1%)	265.7 ± 5.9	0.351 ± 0.041
NPs unwashed	Milli-Q water	236.4 ± 1.2	0.325 ± 0.006

**Table 2:** Zeta potential, mobility, and conductivity determination in PET NPs at different stages of purification.

	NPs Washed (Dispersed in Milli-Q Water)	NPs Unwashed (Dispersed in Milli-Q Water)	NPs Washed (Dispersed in 0.5% SDS)
Zeta potential (mV)	−34.93 ± 1.18	−42.10 ± 1.30	−63.53 ± 1.47
Mobility (µmcm/V)	−2.739 ± 0.094	−3.301 ± 0.100	−4.980 ± 0.114
Conductivity (mS/cm)	0.0205 ± 0.0001	0.0287 ± 0.0105	0.9710 ± 0.0301

## Data Availability

Data are contained within the article and supplementary material.

## Supplementary Materials

The following supporting information can be downloaded at https://www.mdpi.com/article/10.3390/polym15244703/s1, Figure S1: Overall scheme of nPET production procedure; Figure S2: Schematic illustration of washing and size separation processes; Figures S3, S7, S9: Size distributions (by intensity percentage) of NPs washed suspended in different dispersants; Figures S4, S8, S10: Size distributions (by number percentage) of NPs washed suspended in different dispersants; Table S1: Hydrodynamic diameter (d.nm) and polydispersity index of NPs washed obtained from combined fractions; Figure S5: Hydrodynamic diameter (d.nm) of NPs washed obtained from combined fractions; Figure S6: Polydispersity index of NPs washed obtained from combined fractions; Figure S11: Relative quantification of SDS contamination in relation to PET signal in NMR; Figure S12: ^1^H NMR spectrum (400 MHz) of trifluoroacetic acid (TFA) recording with addition of deuterated chloroform (CDCl_3_) in the ratio 4:1 (*v,v, respectively*); Figure S13: ^1^H NMR spectrum (400 MHz) of PET pellet (starting material) recorded in the mixture of TFA and CDCl_3_ in the ratio 4:1 (*v/v*); Figure S14: ^1^H NMR spectrum (400 MHz) of sodium dodecyl sulfate (SDS) recorded in the mixture of TFA and CDCl_3_ in the ratio 4:1 (*v/v*); Table S2: P-Values for hemolysis experiment for Donor 1; Table S3: P-Values for hemolysis experiment for Donor 2; Table S4: P-Values for hemolysis experiment for Donor 3; Table S5: P-Values for hemolysis experiment in Ringer's solution. References [37,38,39] are cited in the supplementary materials.
