# Supplementary material for: Characterization of Nanoprecipitated PET Nanoplastics by 1H NMR and Impact of Residual Ionic Surfactant on Viability of Human Primary Mononuclear Cells and Hemolysis of Erythrocytes
Source: Polymers (Basel). 2023 Dec 13;15(24):4703. doi: 10.3390/polym15244703 (PMC10747210; doi:10.3390/polym15244703)
Supplement: Supplementary file 1 [file polymers-15-04703-s001.zip › polymers-2723046-supplementary.pdf]

Supplementary material

Characterization of nanoprecipitated PET nanoplastics by ^{1}H NMR and impact of residual ionic surfactant on viability of human primary mononuclear cells and hemolysis of erythrocytes

Milica Djapovic [1], Danijela Apostolovic [2], Vojislava Postic [1], Tamara Lujic [1], Vesna Jovanovic [1], Dragana Stanic-Vucinic [1], Marianne van Hagec[2], Veselin Maslak[1*] and Tanja Cirkovic Velickovic [1,3,*]

[1]University of Belgrade - Faculty of Chemistry, Studentski trg 12-16, 11158 Belgrade, Serbia

[2]Immunology and Allergy Division, Center for Molecular Medicine, Department of Medicine Solna, Karolinska Institutet, Stockholm, Sweden

[3]Serbian Academy of Sciences and Arts, Knez Mihajlova 35, 11102 Belgrade, Serbia

***Corresponding authors**

E-mail address: tcirkov@chem.bg.ac.rs (T. Cirkovic Velickovic); vmaslak@chem.bg.ac.rs (V. Maslak)

Preparation of PET NPs

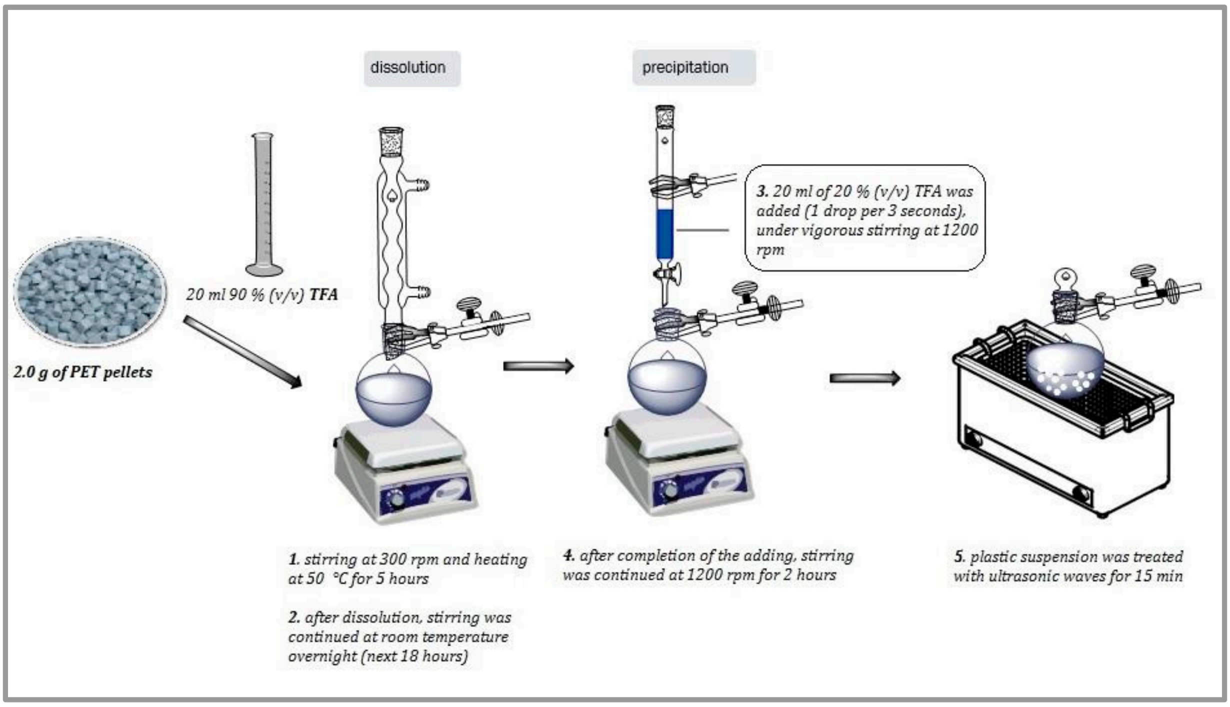

Figure S1. Overall scheme of nPET production procedure. First, 2.0 g of PET pellets was dissolved in 20.0 mL of TFA in Mili-Q water *(v/v)* with stirring at 300 rpm for five hours at 50 °C and next 18h at room temperature. Then, 20 mL of 20% TFA in Milli-Q water *(v/v)* was added dropwise during 110 min (1 drop of 10 μL per 3 s), under vigorous stirring at 1200 rpm using a dropping funnel. Stirring of suspension was continued for an additional 2 hours, before being sonicated in an ultrasonic bath for 15 min.

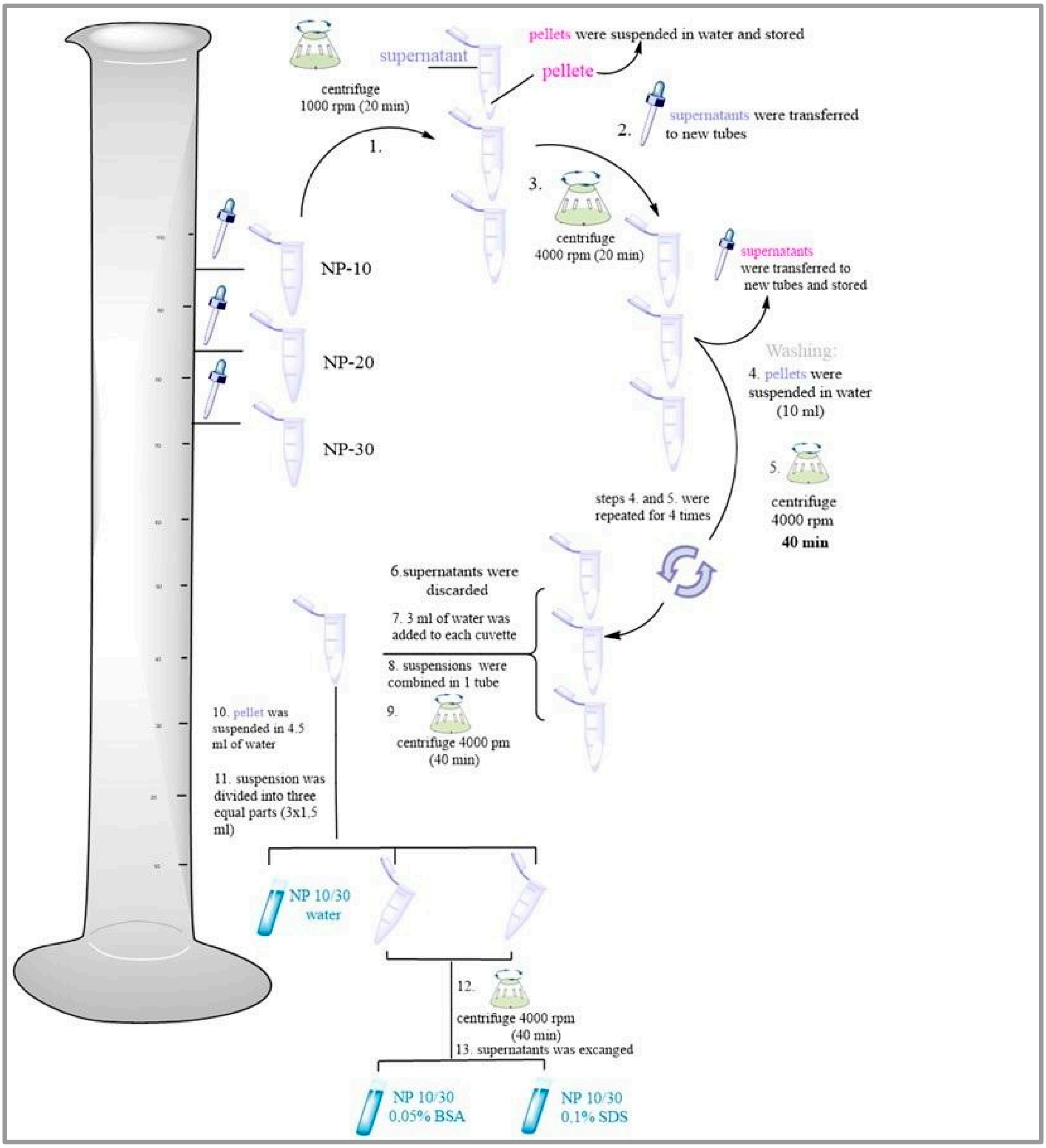

Figure S2. Schematic illustration of washing and size separation processes of the first three fractions (each 10 mL), denoted NP-10, NP-20 and NP-30.

Characterization of PET NPs

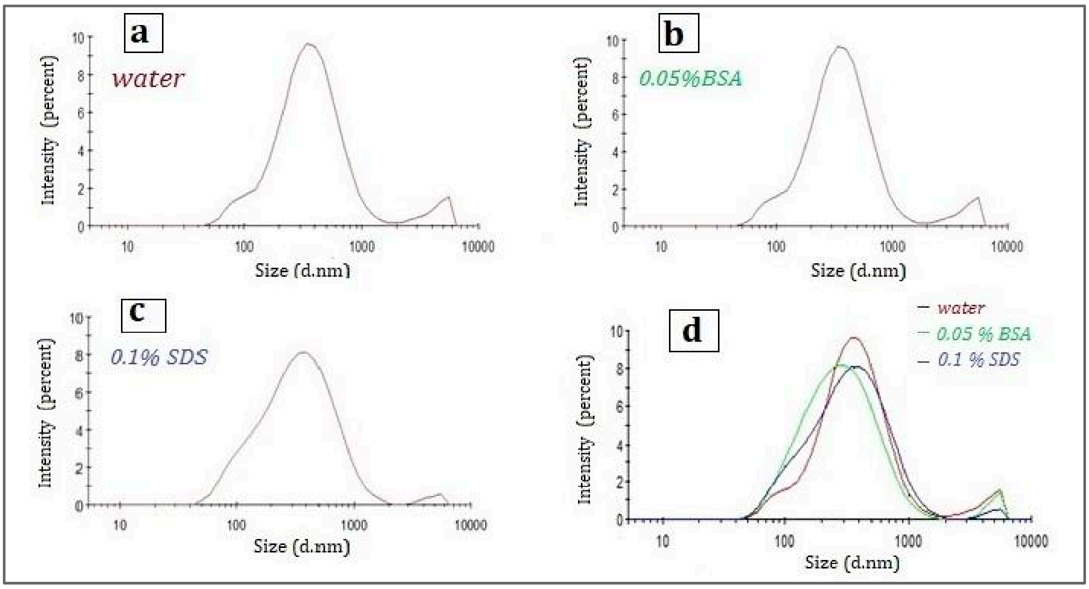

Figure S3. Size distributions (by **intensity percentage**) of **NPs washed** suspended in different dispersants ((**a**)-water, (**b**)-BSA (0.05%), (**c**)-SDS (0.1 %)); (**d**)-combined graphs of different dispersants. Size distributions are shown for NPs obtained from combined fractions: **NP-10**, **NP-20 and NP-30**.

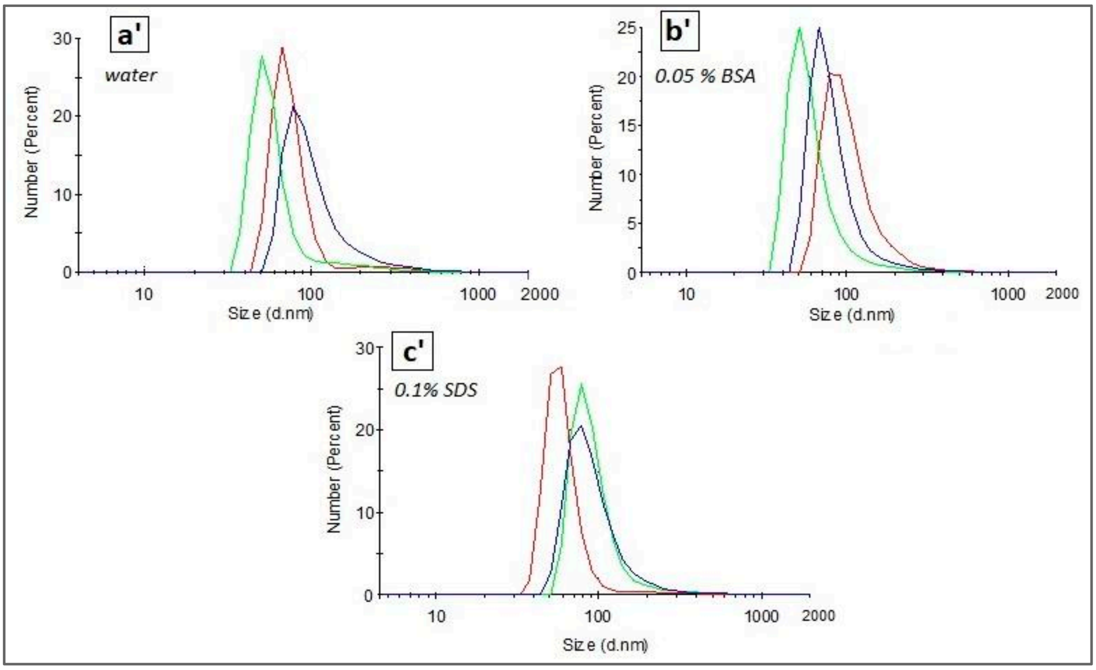

Figure S4. Size distributions (by **number percentage**) of **NPs washed** suspended in different dispersants: ((**a**')-water, (**b**')-BSA (0.05%), (**c**')-SDS (0.1 %)). Distributions are shown for NPs obtained from combined fractions: **NP-10, NP-20 and NP-30**. Additionally multiple consecutive measurements (lines with different colors) are presented.

Table S1. Hydrodynamic diameter (d.nm) and polydispersity index of **NPs washed** obtained from combined fractions: NP-10, NP-20 and NP-30 (denoted **NP-10+NP-20+NP-30**); NP-40, NP-50 and NP-60 (denoted **NP-40+NP-50+NP-60**); NP-70, NP-80 and NP-90 (denoted **NP-70+NP-80+NP-90**) dispersed in different dispersants: water, 0.05% BSA and 0.1% SDS.

	**dispersant**	**NPS washed**		
		NP-10+NP-20+NP-30	NP-40+NP-50+NP-60	NP-70+NP-80+NP-90
**Z-Average (d.nm)**	water	311.7 ± 4.4	182.3 ± 3.4	262.5 ± 9.6
	0.05% BSA	243.5 ± 1.3	264.6 ± 7.2	230.7 ± 1.1
	0.1 % SDS	265.7 ± 5.9	256.8 ± 3.3	215.6 ± 1.6
**Polydispersity index**	water	0.375 ± 0.026	0.309 ± 0.034	0.540 ± 0.073
	0.05% BSA	0.352 ± 0.037	0.271 ± 0.010	0.269 ± 0.011
	0.1 % SDS	0.351 ± 0.041	0.336 ± 0.026	0.260 ± 0.050

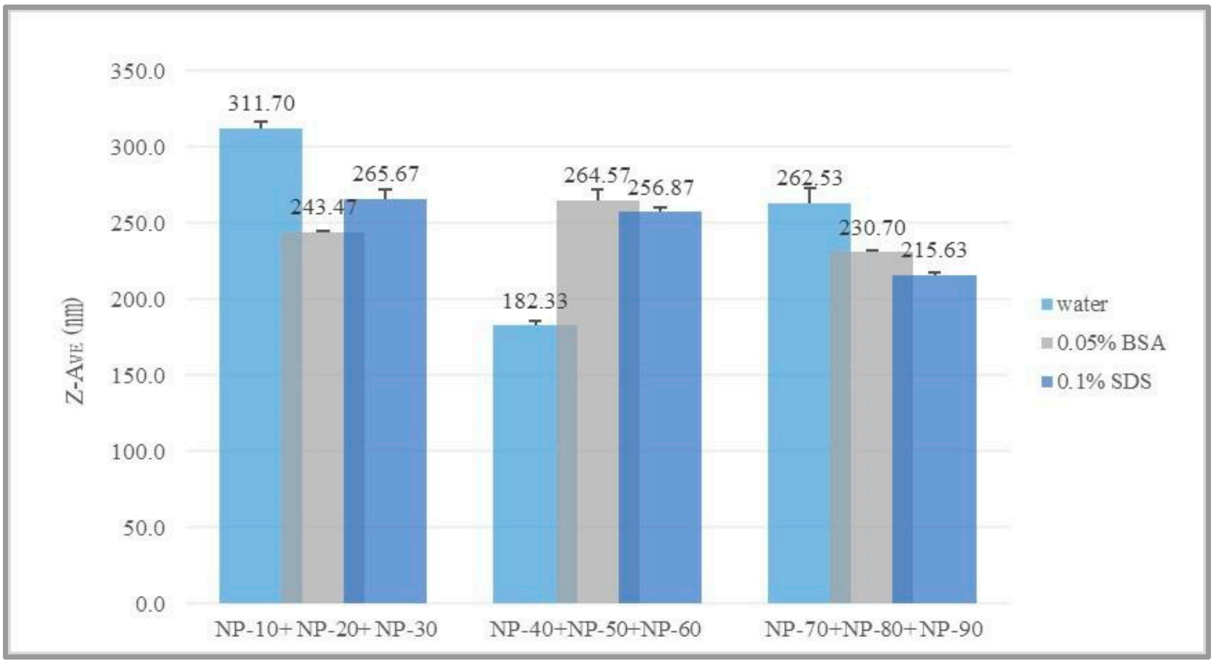

Figure S5. Hydrodynamic diameter (d.nm) of **NPs washed** obtained from combined fractions: NP-10, NP-20 and NP- 30 (denoted **NP-10+NP-20+NP-30**); NP-40, NP-50 and NP-60 (denoted **NP-40+NP-50+NP-60**); NP-70, NP-80 and NP-90 (denoted **NP-70+NP-80+NP-90**) dispersed in different dispersants: water, 0.05% BSA and 0.1% SDS.

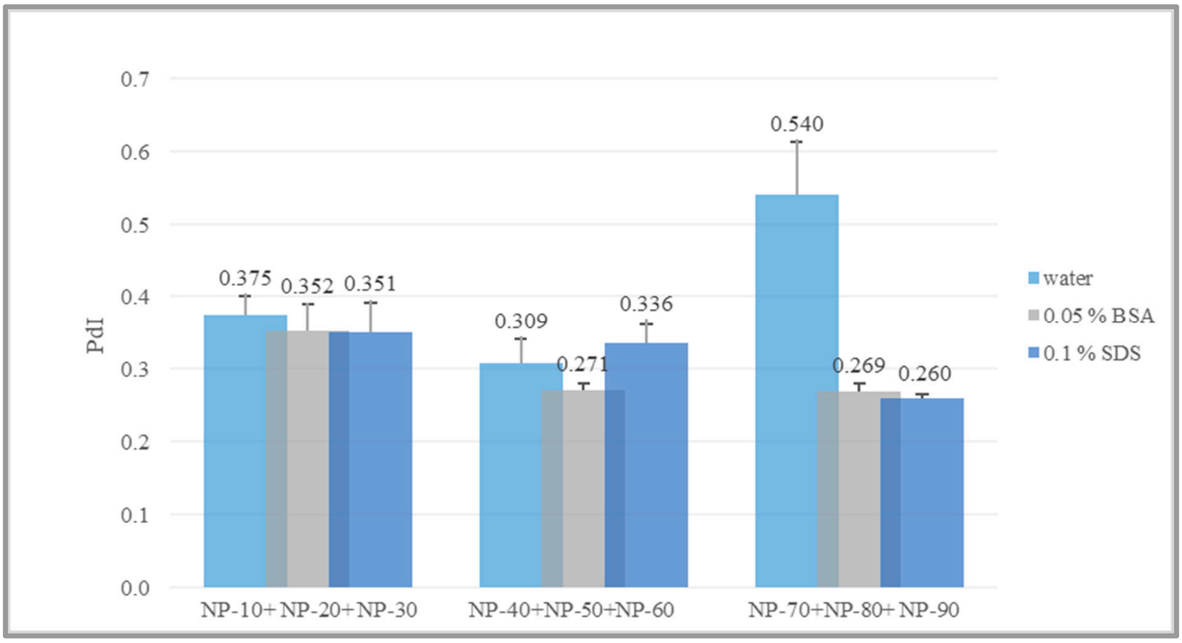

Figure S6. Polydispersity index of **NPs washed** obtained from combined fractions: NP-10, NP-20 and NP-30 (denoted **NP-10+NP-20+NP-30**); NP-40, NP-50 and NP-60 (denoted **NP-40+NP-50+NP-60**); NP-70, NP-80 and NP-90 (denoted **NP-70+NP-80+NP-90**) dispersed in different dispersants: water, 0.05% BSA and 0.1% SDS.

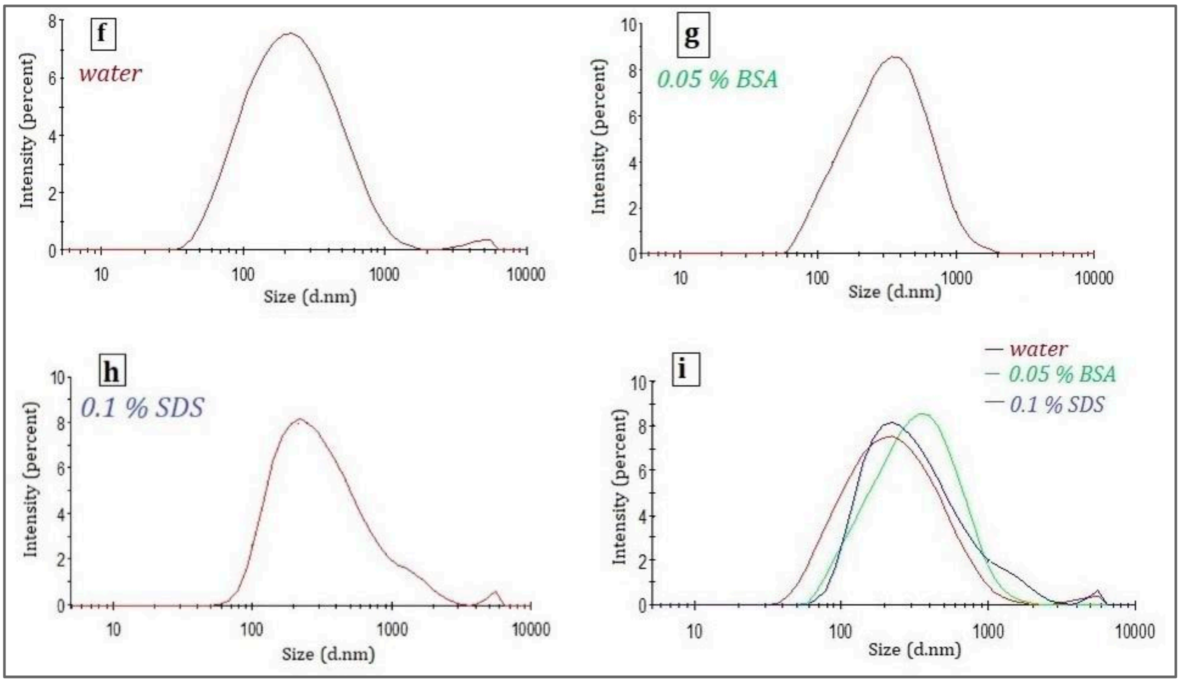

Figure S7. Size distributions (by **intensity percentage**) of **NPs washed** suspended in different dispersants ((**f**)-water, (**g**)-BSA (0.05%), (**h**)-SDS (0.1 %)); (**i**)-combined graphs of different dispersants. Size distributions are shown for NPs obtained from combined fractions: **NP-40**, **NP-50 and NP-60**.

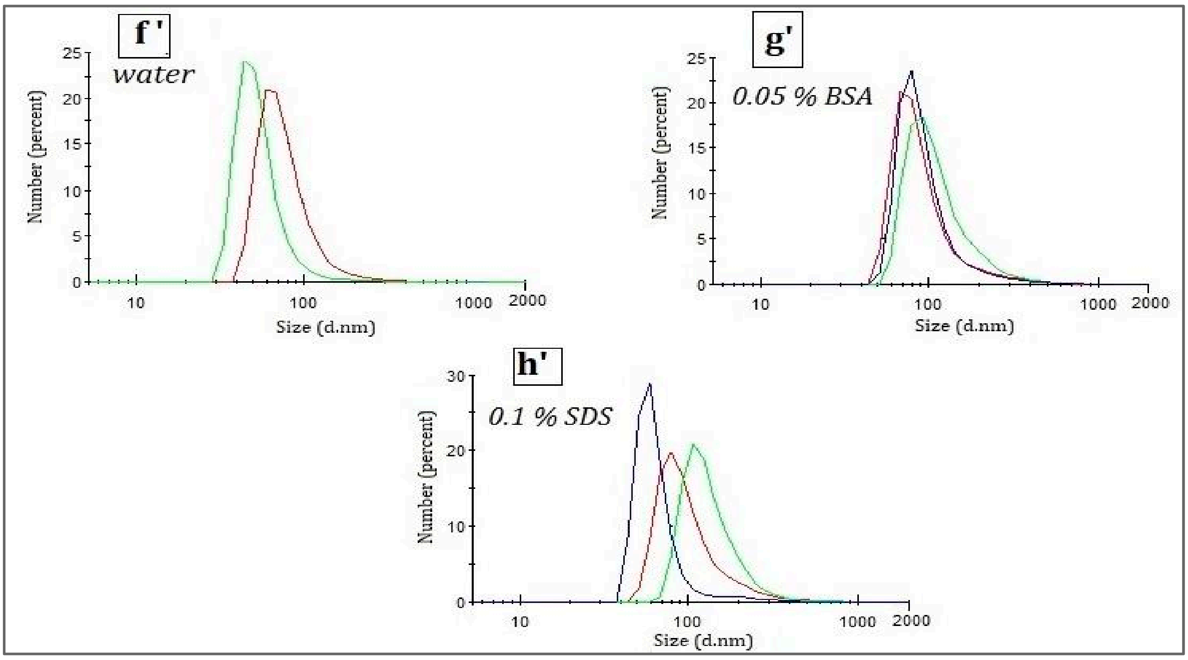

Figure S8. Size distributions (by **number percentage**) of **NPs washed** suspended in different dispersants: ((**f'**)-water, (**g'**)-BSA (0.05%), (**h'**)-SDS (0.1 %)). Distributions are shown for NPs obtained from combined fractions: **NP-40, NP-50 and NP-60**. Additionally multiple consecutive measurements (lines with different colors) are presented.

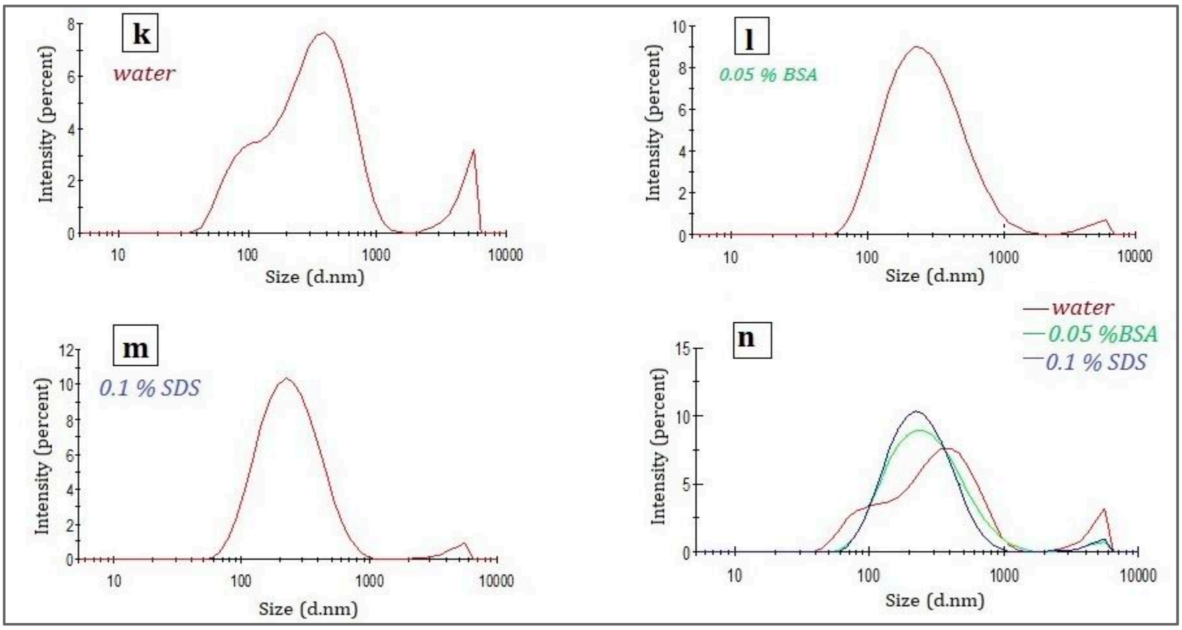

Figure S9. Size distributions (by **intensity percentage**) of **NPs washed** suspended in different dispersants ((**k**)-water, (**l**)-BSA (0.05%), (**m**)-SDS (0.1 %)); (**n**)-combined graphs of different dispersants. Size distributions are shown for NPs obtained from combined fractions: **NP-70**, **NP-80 and NP-90**.

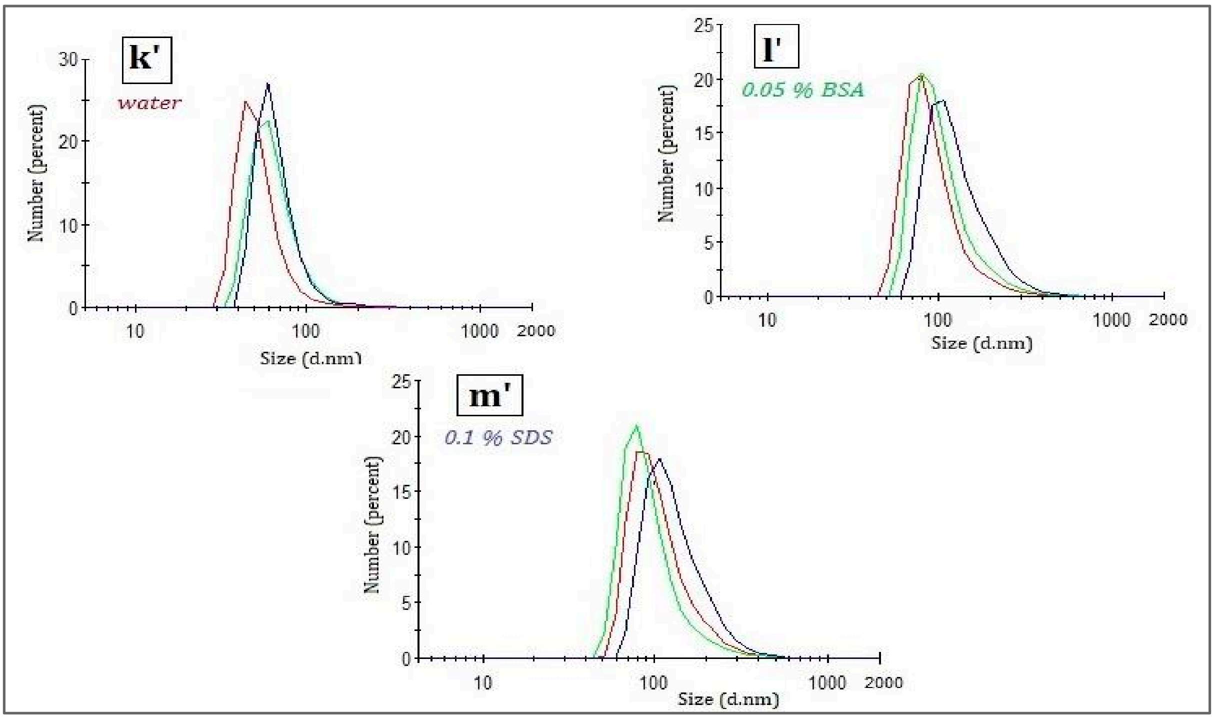

Figure S10. Size distributions (by **number percentage**) of **NPs washed** suspended in different dispersants ((**k'**)-water, (**l'**)-BSA (0.05%), (**m'**)-SDS (0.1 %)). Distributions are shown for NPs obtained from combined fractions: **NP-70, NP-80 and NP-90**. Additionally multiple consecutive measurements (lines with different colors) are presented.

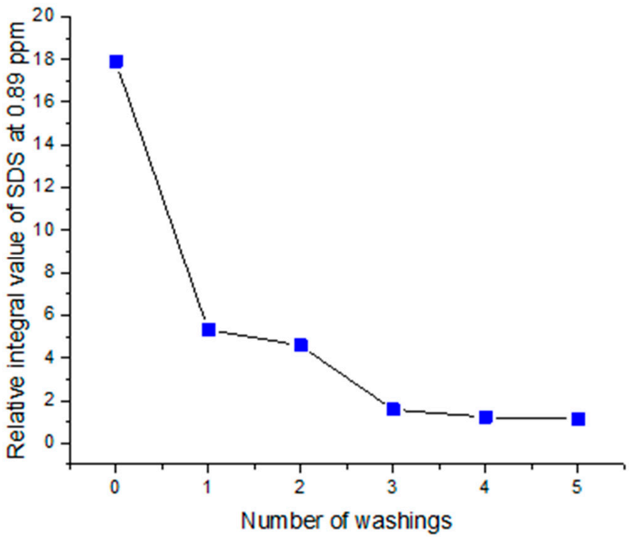

Figure S11. Relative quantification of SDS contamination in relation to PET signal in NMR.

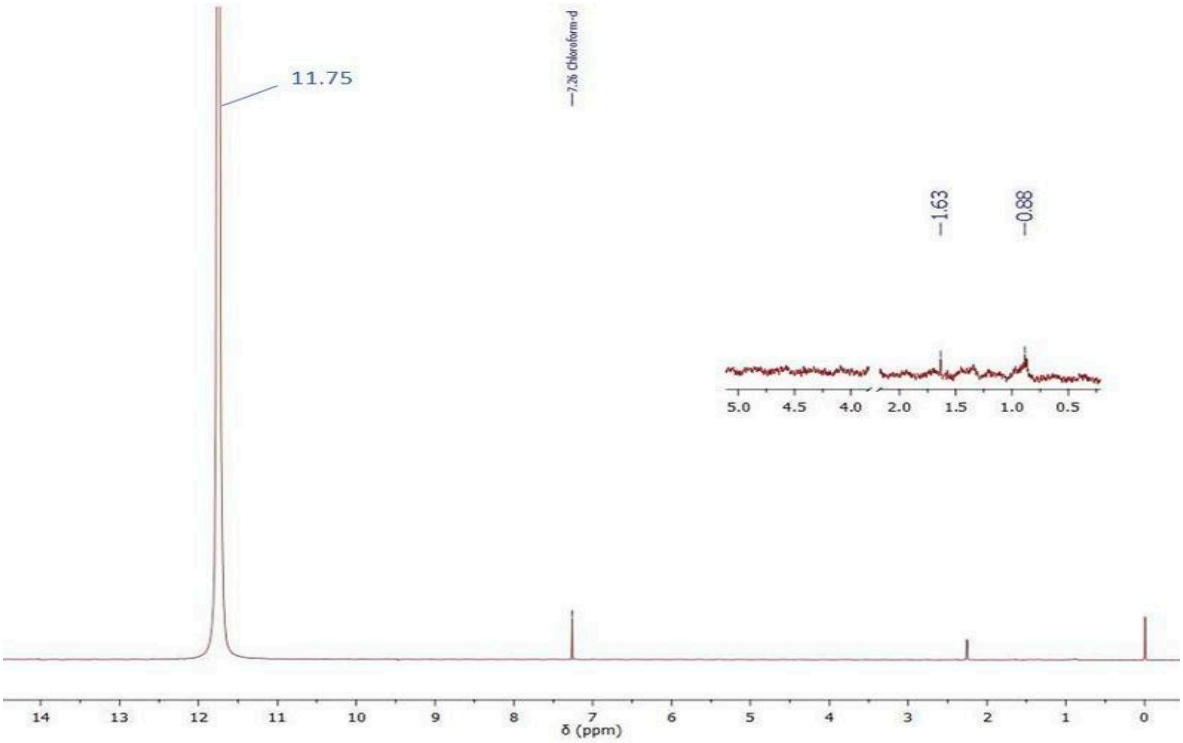

Figure S12. ^{1}H NMR spectrum (400 MHz) of trifluoroacetic acid (TFA) recording with addition of deuterated chloroform ($CDCl_3$) in the ratio 4:1 (*v,v, respectively*). Region importance for process monitoring of washing process of NPs (0.5- 2.0 ppm and 4.0- 5 .0 ppm) is zoomed. Signal at 11.75 ppm originates from TFA; signals with chemical shifts values 0.88 and 1.63 ppm indicate the presence of impurities in very low concentrations.

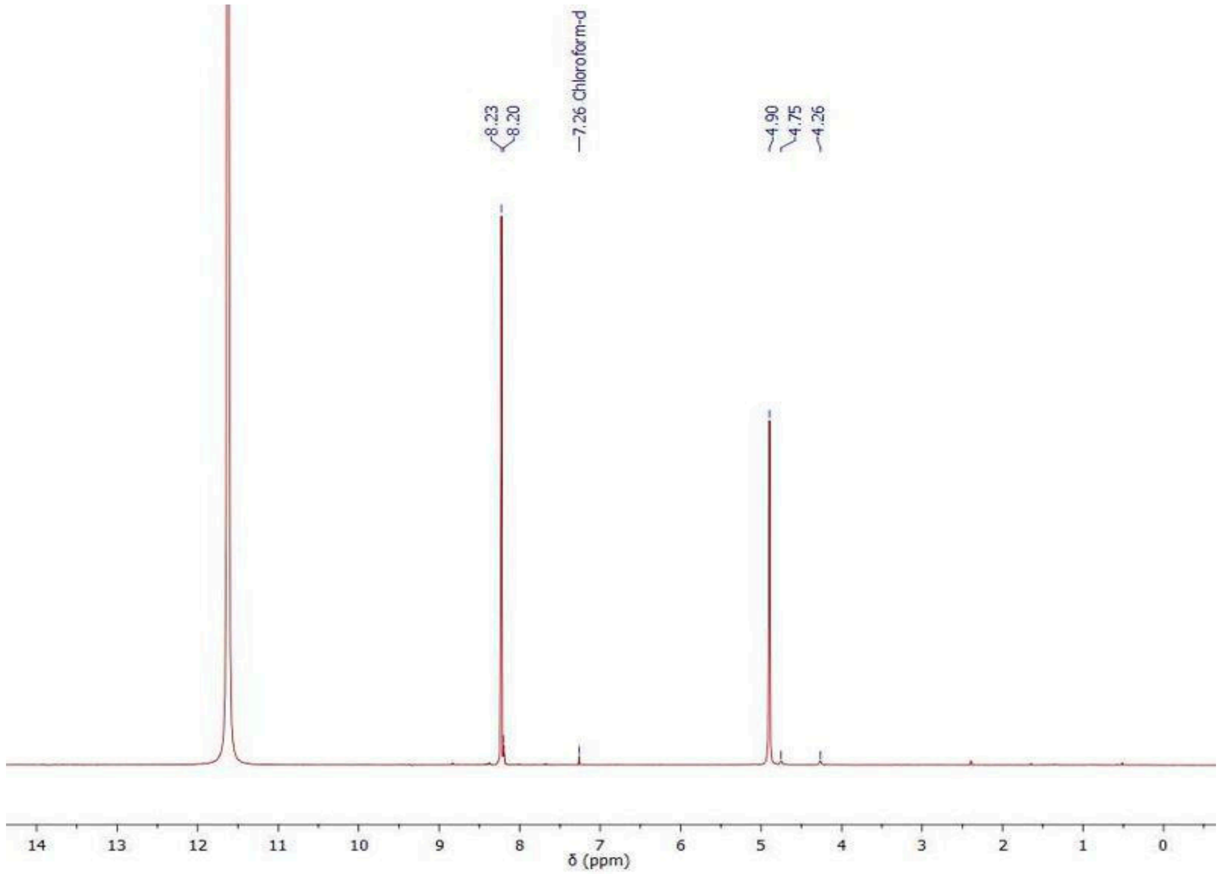

Figure S13. ^{1}H NMR spectrum (400 MHz) of PET pellet (starting material) recorded in the mixture of TFA and $CDCl_3$ in the ratio 4:1 (*v*/*v*). The use of this mixture containing the fluorinated solvent is advantageous, since it allows room temperature spectra [ref. 37 in main text]. Characteristic signals at 8.20- 8.23 (aromatic protons), 4.90 (internal methylene groups), and low intensity signals at 4.75 and 4.26 ppm (the methylene protons adjacent to the hydroxyl end functions) are consistent with reported literature data [ref. 38, 39 ina main text].

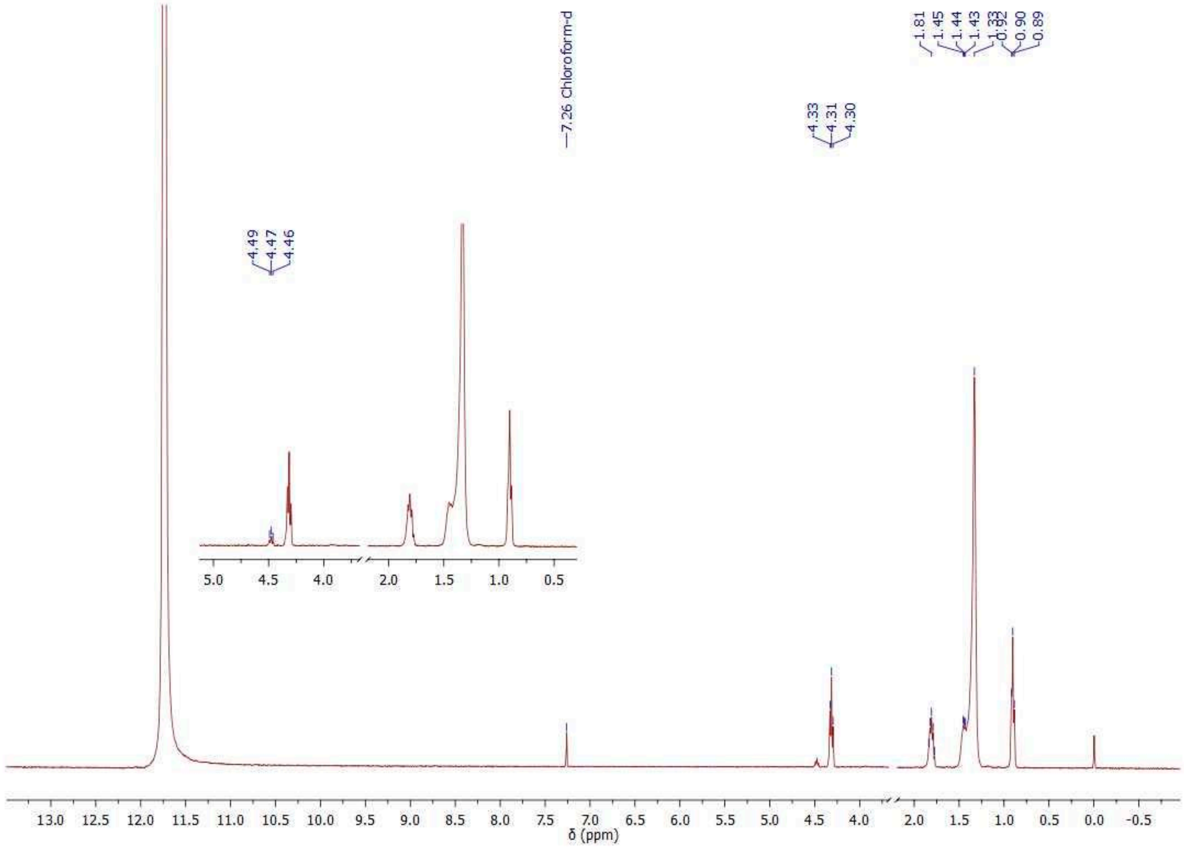

Figure S14. ^{1}H NMR spectrum (400 MHz) of sodium dodecyl sulfate (SDS) recorded in the mixture of TFA and $CDCl_3$ in the ratio 4:1 (*v*/*v*). The peaks at regions 0.89-0.92, 1.33-1.45, 1.81 and 4.30-4.33 ppm originate from SDS. In the magnified part of the spectrum a low intensity signal with chemical shift of 4.46-4.49 ppm is visible. The intensity of this impurity decreases during the washing process of NPs from SDS; already after third washing, this peak is not visible in the NMR spectrum (Figure 1B).

Table S2. P-Values for hemolysis experiment for Donor 1 (Figure 4A)

Samples	Adjusted P Value
0.9% NaCl + NPs unwashed 10 µg/ml vs. 0.9% NaCl + NPs unwashed 1 µg/ml	<0.0001
0.9% NaCl + NPs unwashed 10 µg/ml vs. 0.9% NaCl + NPs unwashed 0.1 µg/ml	<0.0001
0.9% NaCl + NPs unwashed 10 µg/ml vs. 0.9% NaCl + NPs washed 10 µg/ml	<0.0001
0.9% NaCl + NPs unwashed 10 µg/ml vs. 0.9% NaCl + NPs washed 1 µg/ml	<0.0001

0.9% NaCl + NPs unwashed 10 µg/ml vs. 0.9% NaCl + NPs washed 0.1 µg/ml	<0.0001
0.9% NaCl + NPs unwashed 10 µg/ml vs. 0.9% NaCl	<0.0001
0.9% NaCl + NPs unwashed 10 µg/ml vs. 0.98%NaCl	<0.0001
0.9% NaCl + NPs unwashed 10 µg/ml vs. 0.9% NaCl + 0.0003%SDS	<0.0001
0.9% NaCl + NPs unwashed 1 µg/ml vs. 0.9% NaCl + NPs unwashed 0.1 µg/ml	0.0378
0.9% NaCl + NPs unwashed 1 µg/ml vs. 0.9% NaCl + NPs washed 10 µg/ml	0.2876
0.9% NaCl + NPs unwashed 1 µg/ml vs. 0.9% NaCl + NPs washed 1 µg/ml	0.3772
0.9% NaCl + NPs unwashed 1 µg/ml vs. 0.9% NaCl + NPs washed 0.1 µg/ml	0.2876
0.9% NaCl + NPs unwashed 1 µg/ml vs. 0.9% NaCl	0.0698
0.9% NaCl + NPs unwashed 1 µg/ml vs. 0.98%NaCl	0.1396
0.9% NaCl + NPs unwashed 1 µg/ml vs. 0.9% NaCl + 0.0003%SDS	0.0994
0.9% NaCl + NPs unwashed 0.1 µg/ml vs. 0.9% NaCl + NPs washed 10 µg/ml	0.0002
0.9% NaCl + NPs unwashed 0.1 µg/ml vs. 0.9% NaCl + NPs washed 1 µg/ml	0.9124
0.9% NaCl + NPs unwashed 0.1 µg/ml vs. 0.9% NaCl + NPs washed 0.1 µg/ml	0.9611
0.9% NaCl + NPs unwashed 0.1 µg/ml vs. 0.9% NaCl	>0.9999
0.9% NaCl + NPs unwashed 0.1 µg/ml vs. 0.98%NaCl	0.9983
0.9% NaCl + NPs unwashed 0.1 µg/ml vs. 0.9% NaCl + 0.0003%SDS	0.9998
0.9% NaCl + NPs washed 10 µg/ml vs. 0.9% NaCl + NPs washed 1 µg/ml	0.0033
0.9% NaCl + NPs washed 10 µg/ml vs. 0.9% NaCl + NPs washed 0.1 µg/ml	0.0022
0.9% NaCl + NPs washed 10 µg/ml vs. 0.9% NaCl	0.0004
0.9% NaCl + NPs washed 10 µg/ml vs. 0.98%NaCl	0.0009
0.9% NaCl + NPs washed 10 µg/ml vs. 0.9% NaCl + 0.0003%SDS	0.0006
0.9% NaCl + NPs washed 1 µg/ml vs. 0.9% NaCl + NPs washed 0.1 µg/ml	>0.9999
0.9% NaCl + NPs washed 1 µg/ml vs. 0.9% NaCl	0.9803
0.9% NaCl + NPs washed 1 µg/ml vs. 0.98%NaCl	0.9991

0.9% NaCl + NPs washed 1 µg/ml vs. 0.9% NaCl + 0.0003%SDS	0.9947
0.9% NaCl + NPs washed 0.1 µg/ml vs. 0.9% NaCl	0.9947
0.9% NaCl + NPs washed 0.1 µg/ml vs. 0.98%NaCl	>0.9999
0.9% NaCl + NPs washed 0.1 µg/ml vs. 0.9% NaCl + 0.0003%SDS	0.9991
0.9% NaCl vs. 0.98%NaCl	>0.9999
0.9% NaCl vs. 0.9% NaCl + 0.0003%SDS	>0.9999
0.98%NaCl vs. 0.9% NaCl + 0.0003%SDS	>0.9999

Table S3. P-Values for hemolysis experiment for Donor 2 (Figure 4B)

**Samples**	**Adjusted P Value**
0.9% NaCl + NPs unwashed 10 µg/ml vs. 0.9% NaCl + NPs unwashed 1 µg/ml	<0.0001
0.9% NaCl + NPs unwashed 10 µg/ml vs. 0.9% NaCl + NPs unwashed 0.1 µg/ml	<0.0001
0.9% NaCl + NPs unwashed 10 µg/ml vs. 0.9% NaCl + NPs washed 10 µg/ml	<0.0001
0.9% NaCl + NPs unwashed 10 µg/ml vs. 0.9% NaCl + NPs washed 1 µg/ml	<0.0001
0.9% NaCl + NPs unwashed 10 µg/ml vs. 0.9% NaCl + NPs washed 0.1 µg/ml	<0.0001
0.9% NaCl + NPs unwashed 10 µg/ml vs. 0.9% NaCl	<0.0001
0.9% NaCl + NPs unwashed 10 µg/ml vs. 0.98%NaCl	<0.0001
0.9% NaCl + NPs unwashed 10 µg/ml vs. 0.9% NaCl + 0.0003%SDS	<0.0001
0.9% NaCl + NPs unwashed 1 µg/ml vs. 0.9% NaCl + NPs unwashed 0.1 µg/ml	0.0001
0.9% NaCl + NPs unwashed 1 µg/ml vs. 0.9% NaCl + NPs washed 10 µg/ml	<0.0001
0.9% NaCl + NPs unwashed 1 µg/ml vs. 0.9% NaCl + NPs washed 1 µg/ml	0.0381
0.9% NaCl + NPs unwashed 1 µg/ml vs. 0.9% NaCl + NPs washed 0.1 µg/ml	0.0004
0.9% NaCl + NPs unwashed 1 µg/ml vs. 0.9% NaCl	0.0003
0.9% NaCl + NPs unwashed 1 µg/ml vs. 0.98%NaCl	<0.0001
0.9% NaCl + NPs unwashed 1 µg/ml vs. 0.9% NaCl + 0.0003%SDS	<0.0001

0.9% NaCl + NPs unwashed 0.1 µg/ml vs. 0.9% NaCl + NPs washed 10 µg/ml	<0.0001
0.9% NaCl + NPs unwashed 0.1 µg/ml vs. 0.9% NaCl + NPs washed 1 µg/ml	0.1761
0.9% NaCl + NPs unwashed 0.1 µg/ml vs. 0.9% NaCl + NPs washed 0.1 µg/ml	0.9995
0.9% NaCl + NPs unwashed 0.1 µg/ml vs. 0.9% NaCl	0.9999
0.9% NaCl + NPs unwashed 0.1 µg/ml vs. 0.98%NaCl	0.9999
0.9% NaCl + NPs unwashed 0.1 µg/ml vs. 0.9% NaCl + 0.0003%SDS	0.4936
0.9% NaCl + NPs washed 10 µg/ml vs. 0.9% NaCl + NPs washed 1 µg/ml	<0.0001
0.9% NaCl + NPs washed 10 µg/ml vs. 0.9% NaCl + NPs washed 0.1 µg/ml	<0.0001
0.9% NaCl + NPs washed 10 µg/ml vs. 0.9% NaCl	<0.0001
0.9% NaCl + NPs washed 10 µg/ml vs. 0.98%NaCl	<0.0001
0.9% NaCl + NPs washed 10 µg/ml vs. 0.9% NaCl + 0.0003%SDS	<0.0001
0.9% NaCl + NPs washed 1 µg/ml vs. 0.9% NaCl + NPs washed 0.1 µg/ml	0.4269
0.9% NaCl + NPs washed 1 µg/ml vs. 0.9% NaCl	0.3649
0.9% NaCl + NPs washed 1 µg/ml vs. 0.98%NaCl	0.0755
0.9% NaCl + NPs washed 1 µg/ml vs. 0.9% NaCl + 0.0003%SDS	0.0027
0.9% NaCl + NPs washed 0.1 µg/ml vs. 0.9% NaCl	>0.9999
0.9% NaCl + NPs washed 0.1 µg/ml vs. 0.98%NaCl	0.9735
0.9% NaCl + NPs washed 0.1 µg/ml vs. 0.9% NaCl + 0.0003%SDS	0.2141
0.9% NaCl vs. 0.98%NaCl	0.9870
0.9% NaCl vs. 0.9% NaCl + 0.0003%SDS	0.2582
0.98%NaCl vs. 0.9% NaCl + 0.0003%SDS	0.7696

Table S4. P-Values for hemolysis experiment for Donor 3 (Figure 4C)

**Samples**	**Adjusted P Value**
0.9% NaCl + NPs unwashed 10 µg/ml vs. 0.9% NaCl + NPs unwashed 1 µg/ml	<0.0001

0.9% NaCl + NPs unwashed 10 µg/ml vs. 0.9% NaCl + NPs unwashed 0.1 µg/ml	<0.0001
0.9% NaCl + NPs unwashed 10 µg/ml vs. 0.9% NaCl + NPs washed 10 µg/ml	0.3980
0.9% NaCl + NPs unwashed 10 µg/ml vs. 0.9% NaCl + NPs washed 1 µg/ml	<0.0001
0.9% NaCl + NPs unwashed 10 µg/ml vs. 0.9% NaCl + NPs washed 0.1 µg/ml	<0.0001
0.9% NaCl + NPs unwashed 10 µg/ml vs. 0.9% NaCl	<0.0001
0.9% NaCl + NPs unwashed 10 µg/ml vs. 0.98%NaCl	<0.0001
0.9% NaCl + NPs unwashed 10 µg/ml vs. 0.9% NaCl + 0.0003%SDS	<0.0001
0.9% NaCl + NPs unwashed 1 µg/ml vs. 0.9% NaCl + NPs unwashed 0.1 µg/ml	0.0009
0.9% NaCl + NPs unwashed 1 µg/ml vs. 0.9% NaCl + NPs washed 10 µg/ml	<0.0001
0.9% NaCl + NPs unwashed 1 µg/ml vs. 0.9% NaCl + NPs washed 1 µg/ml	0.2124
0.9% NaCl + NPs unwashed 1 µg/ml vs. 0.9% NaCl + NPs washed 0.1 µg/ml	0.0058
0.9% NaCl + NPs unwashed 1 µg/ml vs. 0.9% NaCl	0.0009
0.9% NaCl + NPs unwashed 1 µg/ml vs. 0.98%NaCl	0.2962
0.9% NaCl + NPs unwashed 1 µg/ml vs. 0.9% NaCl + 0.0003%SDS	0.3450
0.9% NaCl + NPs unwashed 0.1 µg/ml vs. 0.9% NaCl + NPs washed 10 µg/ml	<0.0001
0.9% NaCl + NPs unwashed 0.1 µg/ml vs. 0.9% NaCl + NPs washed 1 µg/ml	0.4545
0.9% NaCl + NPs unwashed 0.1 µg/ml vs. 0.9% NaCl + NPs washed 0.1 µg/ml	0.9990
0.9% NaCl + NPs unwashed 0.1 µg/ml vs. 0.9% NaCl	>0.9999
0.9% NaCl + NPs unwashed 0.1 µg/ml vs. 0.98%NaCl	0.3450
0.9% NaCl + NPs unwashed 0.1 µg/ml vs. 0.9% NaCl + 0.0003%SDS	0.2962
0.9% NaCl + NPs washed 10 µg/ml vs. 0.9% NaCl + NPs washed 1 µg/ml	<0.0001
0.9% NaCl + NPs washed 10 µg/ml vs. 0.9% NaCl + NPs washed 0.1 µg/ml	<0.0001
0.9% NaCl + NPs washed 10 µg/ml vs. 0.9% NaCl	<0.0001
0.9% NaCl + NPs washed 10 µg/ml vs. 0.98%NaCl	<0.0001
0.9% NaCl + NPs washed 10 µg/ml vs. 0.9% NaCl + 0.0003%SDS	<0.0001

0.9% NaCl + NPs washed 1 µg/ml vs. 0.9% NaCl + NPs washed 0.1 µg/ml	0.8485
0.9% NaCl + NPs washed 1 µg/ml vs. 0.9% NaCl	0.4545
0.9% NaCl + NPs washed 1 µg/ml vs. 0.98%NaCl	>0.9999
0.9% NaCl + NPs washed 1 µg/ml vs. 0.9% NaCl + 0.0003%SDS	>0.9999
0.9% NaCl + NPs washed 0.1 µg/ml vs. 0.9% NaCl	0.9990
0.9% NaCl + NPs washed 0.1 µg/ml vs. 0.98%NaCl	0.7503
0.9% NaCl + NPs washed 0.1 µg/ml vs. 0.9% NaCl + 0.0003%SDS	0.6940
0.9% NaCl vs. 0.98%NaCl	0.3450
0.9% NaCl vs. 0.9% NaCl + 0.0003%SDS	0.2962
0.98%NaCl vs. 0.9% NaCl + 0.0003%SDS	>0.9999

Table S5. P-Values for hemolysis experiment in Ringer solution (Figure 5)

Samples	Adjusted P Value
Ringer vs. Ringer Glc HSA	<0.0001
Ringer vs. NPs unwashed 10 µg/mL + Ringer	<0.0001
Ringer vs. NPs washed 10 µg/mL + Ringer	<0.0001
Ringer vs. NPs unwashed 10 µg/mL + Ringer Glc HSA	0.0001
Ringer vs. NPs washed 10 µg/mL + Ringer Glc HSA	0.0003
Ringer Glc HSA vs. NPs unwashed 10 µg/mL + Ringer	<0.0001
Ringer Glc HSA vs. NPs washed 10 µg/mL + Ringer	<0.0001
Ringer Glc HSA vs. NPs unwashed 10 µg/mL + Ringer Glc HSA	0.0023
Ringer Glc HSA vs. NPs washed 10 µg/mL + Ringer Glc HSA	0.0011
NPs unwashed 10 µg/mL + Ringer vs. NPs washed 10 µg/mL + Ringer	<0.0001
NPs unwashed 10 µg/mL + Ringer vs. NPs unwashed 10 µg/mL + Ringer Glc HSA	<0.0001
NPs unwashed 10 µg/mL + Ringer vs. NPs washed 10 µg/mL + Ringer Glc HSA	<0.0001

NPs washed 10 µg/mL + Ringer vs. NPs unwashed 10 µg/mL + Ringer Glc HSA	<0.0001
NPs washed 10 µg/mL + Ringer vs. NPs washed 10 µg/mL + Ringer Glc HSA	<0.0001
NPs unwashed 10 µg/mL + Ringer Glc HSA vs. NPs washed 10 µg/mL + Ringer Glc HSA	0.9997
